# Comparator Event Rates, Baseline Activity, and Operational Selection Pressure in Relapsing Multiple Sclerosis Trials: A Meta‐Epidemiological Analysis

**DOI:** 10.1111/ene.70781

**Published:** 2026-10-10

**Authors:** Steffen Pfeuffer, Lars Masanneck, Marc Pawlitzki, Hans‐Peter Hartung

**Affiliations:** ^1^ Department of Neurology Justus Liebig University Giessen Giessen Germany; ^2^ Department of Neurology, Medical Faculty Heinrich Heine University Düsseldorf Düsseldorf Germany; ^3^ Department of Neurology University Hospital Münster Münster Germany; ^4^ Brain and Mind Centre University of Sydney Sydney Australia; ^5^ Department of Neurology Palacký University Olomouc Olomouc Czech Republic

**Keywords:** clinical trials, drug approval, multiple sclerosis, patient selection, Phase III, relapsing–remitting, treatment outcome

## Abstract

**Background:**

Reference‐arm annualized relapse rates (ARRs) in relapsing multiple sclerosis trials have declined. We examined whether baseline disease activity and the treatment environment during recruitment changed alongside this trend.

**Methods:**

We analyzed 15 treated‐reference arms from nine Phase 3 programs. ARR was related to recruitment era, prior‐year relapse activity, MRI markers, and an Operational Selection Pressure Index (OSPI) combining recruitment geography with time since ocrelizumab approval. Associations were descriptive; disability outcomes were audited separately.

**Results:**

Reference‐arm ARR declined from 0.370 in TEMSO and 0.320 in TOWER to 0.109–0.140 in BTK inhibitor‐era programs (*ρ* = −0.906; 95% CI, −0.985 to −0.279; *n* = 15). Prior‐year relapse activity correlated with ARR (*ρ* = 0.739; 95% CI, 0.158 to 0.928; *n* = 13) and declined over time (*ρ* = −0.725; 95% CI, −0.965 to −0.157; *n* = 13). MRI findings were inconsistent. OSPI clustered at 0–7.3 in six programs and 65.2–80.5 in BTK inhibitor‐era programs and correlated inversely with ARR (*ρ* = −0.892; 95% CI, −1.000 to −0.569; *n* = 9). The association persisted across sensitivities but was negligible after adjustment for recruitment midpoint (partial *ρ* = −0.020). Disability trends depended on reporting metric and effective follow‐up.

**Conclusions:**

Lower baseline relapse activity accompanied declining event yield. OSPI made a treatment‐ecosystem transition measurable but could not be separated from calendar era or establish mechanism. Trials require conservative event assumptions, harmonized reporting, and validation using screening and country‐ or period‐specific outcomes.

## Graphical abstract

Across 15 treated‐reference arms from nine phase 3 relapsing multiple sclerosis programs, reference‐arm relapse rates declined alongside baseline prior‐year relapse activity. A geography‐weighted Operational Selection Pressure Index, reflecting ocrelizumab approval maturity during recruitment, was associated with lower program‐level relapse rates but requires prospective validation. These findings support conservative event assumptions and recruitment‐aware trial design in contemporary treatment settings.

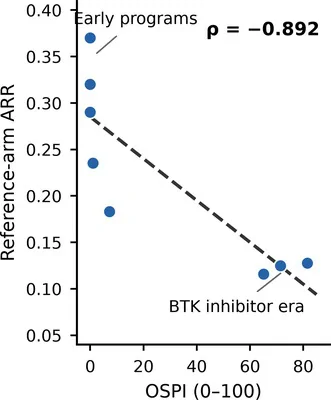

## 1 Introduction

Low relapse event rates have become a central design problem in relapsing multiple sclerosis (MS) trials. Systematic reviews and meta‐regressions documented declining placebo‐arm annualized relapse rates (ARRs) and disability‐progression rates over time and related this trend to baseline relapse activity, study duration, eligibility criteria, diagnostic‐era changes, and regression to the mean [1, 2, 3, 4, 5, 6]. Predictive modeling has further shown that interpretation depends on the randomized population's baseline risk [7]. A lower‐risk population can provide an internally valid estimate for its defined target population, yet an unexpectedly low reference‐arm ARR can render planning assumptions inaccurate and limit inference for patients at higher inflammatory risk.

The problem now extends to active‐comparator programs. Observational and registry studies support earlier high‐efficacy disease‐modifying therapy (DMT), with better long‐term outcomes than delayed escalation [8, 9]. Substantial off‐label rituximab use in Sweden also showed how anti‐CD20 therapy could enter routine MS care before formal MS‐specific approval [10].

In this environment, randomization to platform or moderate‐efficacy therapy can become unattractive for patients with highly active disease. Protocol criteria define eligibility, but unmeasured clinician and patient decisions influence who remains available before randomization. We call this trial‐level context “operational selection pressure”: patients most likely to contribute relapse endpoints may also be least willing, or least appropriate, to accept a lower‐efficacy comparator. This tension becomes consequential where high‐efficacy therapy shapes routine care while pivotal programs must remain applicable to contemporary practice.

Interpretation is constrained by baseline reporting. Relapse history is clinically intuitive but coarse. MRI activity is biologically close to inflammatory relapse risk, and trial‐level effects on MRI lesions correlate with relapse effects [11, 12]. Yet public baseline tables often provide only selected MRI summaries; binary gadolinium‐enhancing (Gd+) positivity, for example, cannot distinguish minimal from extensive enhancement.

The teriflunomide‐comparator era makes the problem unusually visible. After evolutionRMS did not demonstrate superiority, a retrospective comparison with earlier Phase 3 programs highlighted its unexpectedly low teriflunomide ARR [13]. The display identified the phenomenon without explaining why increasingly quiet reference arms had emerged; subsequent BTK inhibitor reports and syntheses reinforced the design challenge [14, 15, 16].

We analyzed public RMS trial data to determine whether reference‐arm ARR declined over enrollment era; whether baseline relapse and MRI activity tracked that decline; and whether recruitment timing, geography, and anti‐CD20 approval could be translated into an interpretable measure of operational selection pressure.

## 2 Methods

### 2.1 Data Sources

We conducted a descriptive meta‐epidemiological analysis of selected RMS treated‐reference arms: teriflunomide 14 mg in TEMSO and TOWER [17, 18]; interferon beta‐1a in OPERA I/II, included because this program introduced ocrelizumab into pivotal RMS trials [19]; and teriflunomide 14 mg in OPTIMUM [20], ASCLEPIOS I/II [21], ULTIMATE I/II [22], evolutionRMS 1/2 [13], GEMINI 1/2 [14], and FENhance 1/2 [15].

The TEMSO and TOWER placebo arms were retained only as historical context for Figure 1 and excluded from all treated‐reference trend, baseline‐activity, and OSPI analyses.

**FIGURE 1:**
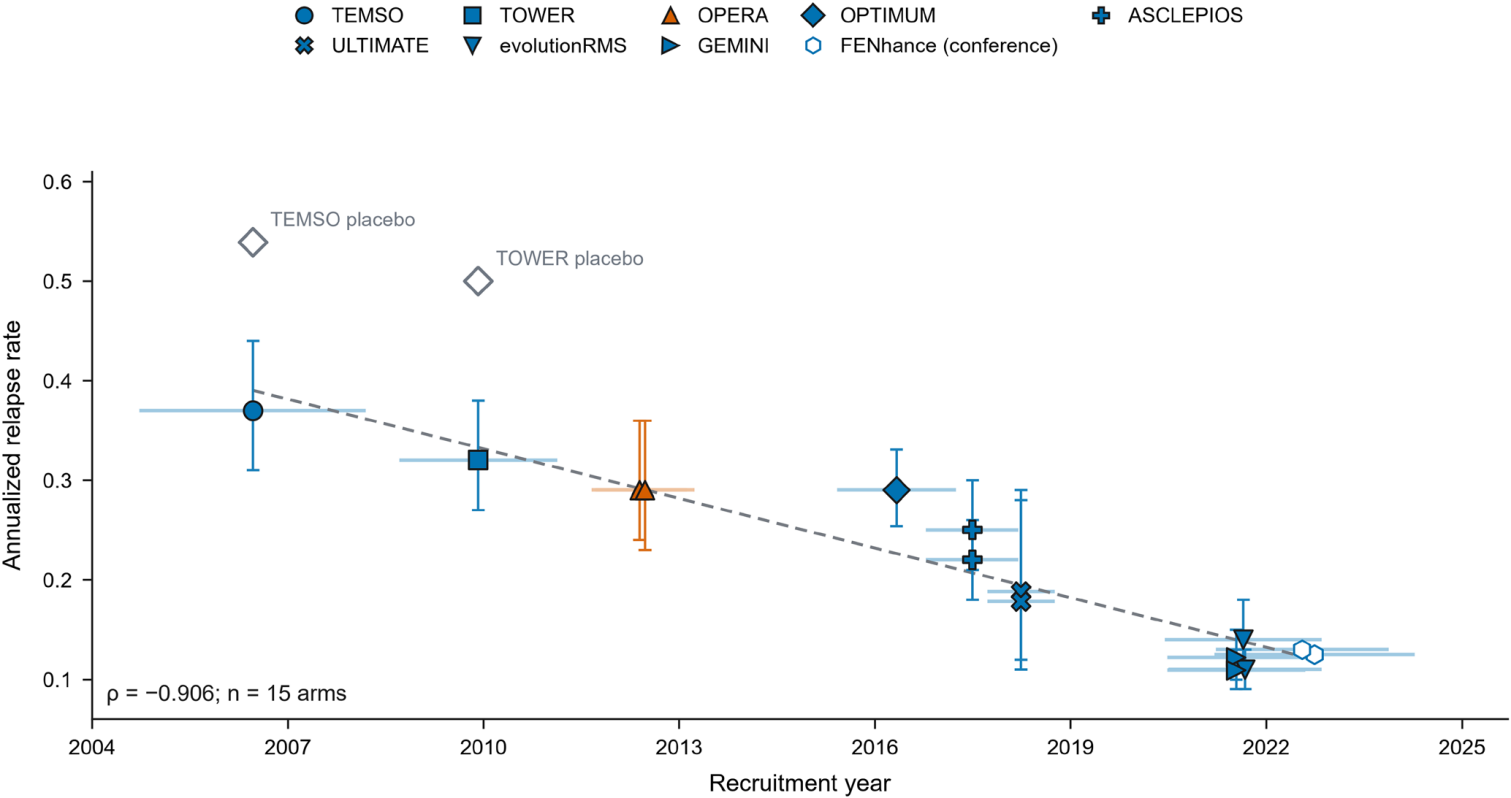
Reference‐arm annualized relapse rates across recruitment eras. Points show ARR at recruitment midpoint; horizontal bars show recruitment windows, and vertical bars show source‐reported 95% CIs. The dashed least‐squares line and Spearman rank correlation include the TEMSO and TOWER teriflunomide 14‐mg arms, OPERA I/II interferon beta‐1a comparator arms, and teriflunomide active‐comparator arms from OPTIMUM, ASCLEPIOS I/II, ULTIMATE I/II, evolutionRMS 1/2, GEMINI 1/2, and FENhance 1/2. Blue markers denote teriflunomide reference arms, orange markers denote OPERA interferon beta‐1a arms, and marker shape identifies the trial program. FENhance 1/2 are shown with open hexagons; their ARR point estimates were reported numerically, whereas confidence limits were available only graphically. Approximate graph‐read limits are provided in Table S1 but are not displayed here. TEMSO and TOWER placebo arms are shown as hollow gray diamonds for historical context only and are excluded from the fitted line and correlation. ARR, annualized relapse rate; CI, confidence interval.

Sources included main reports and supplements, study‐specific MRI or subgroup reports, ClinicalTrials.gov, the European Union Clinical Trials Register, regulatory and health technology assessment reviews, and public Phase 3 material. Registries supplied enrollment dates, locations, and posted results [23, 24]. When sources differed, we prioritized peer‐reviewed reports and supplements, followed by registry results, regulatory or health technology assessment documents, and other public sources.

FENhance values came from preliminary conference data [15]. Analyses were repeated without FENhance. Only aggregate public data were used; missing values were recorded as not reported and were not imputed.

### 2.2 Data Extraction

For each arm, we extracted publication and enrollment timing; reference treatment and sample size; ARR and confidence interval; prior‐year relapse activity or relapse history over 1 or 2 years; age, sex, Expanded Disability Status Scale score, disease duration, and prior DMT exposure; baseline Gd + T1 lesion count and positivity; Gd + T1 lesion volume; and T2 lesion volume and count (Tables S1 and S2). We separately extracted 12‐ and 24‐week EDSS‐confirmed disability outcomes (Table S10). Source locators are provided in Table S3.

Recruitment windows were reconstructed from reported randomization or enrollment dates and registry histories. Exact start and end dates or months were available for 10 of 15 arms; a documented registry‐status proxy represented the end date for the other five. Primary completion dates were not used. Because within‐study recruitment curves were unavailable, time analyses used enrollment start, midpoint, and end, with publication year as a sensitivity scale (Table S3).

Gd + positivity was the reported proportion with at least one baseline Gd + lesion or was calculated from the proportion without enhancement. Other MRI measures followed source definitions. Baseline burden was separated from change from baseline, on‐treatment new or enlarging lesions, and cumulative lesion counts. TOWER did not collect MRI endpoints and was excluded from MRI analyses.

We recorded diagnostic criteria, formal activity requirements, MRI alternatives, baseline MRI acquisition, central MRI reading, and objective relapse confirmation or independent adjudication when documented. We also extracted absolute reference‐arm ARRs in mutually exclusive subgroups defined by MRI activity, relapse history, Expanded Disability Status Scale score, or treatment history. These design features and subgroup estimates were not included in OSPI.

EDSS‐based 12‐week confirmed disability progression or worsening (CDP12) was the principal disability outcome; 24‐week confirmation (CDP24) was summarized where observed. The approximately 2‐year series preferred fixed‐time Kaplan–Meier estimates at weeks 96–108 or month 24 and otherwise used fixed‐duration event proportions (Table S10A). Crude proportions and preliminary FENhance EDSS‐only CDP12 were sensitivities; composite cCDP was excluded (Table S10B).

### 2.3 Operational Selection Pressure Index

OSPI was calculated at program level from recruitment timing and geography relative to public ocrelizumab approval. Country weights used participant counts where available; TEMSO and FENhance 1/2 used trial‐specific ClinicalTrials.gov site‐count proxies because country‐level participant counts were unavailable. Arm‐specific country distributions were unavailable, so geography characterized the program‐level recruitment environment.

For country c, program t, recruitment timing s, and maturation horizon H, maturity was m(c,t,s; H) = min[max{Y(t,s) − A(c),0}/H,1], where Y was enrollment start, midpoint, or end year and A was approval year. OSPI was the geography‐weighted mean on a 0–100 scale: OSPI(t,s; H) = 100 × [Σc w(c,t)m(c,t,s; H)]/[Σc w(c,t)], with w denoting country participant counts or, for TEMSO and FENhance, trial‐specific site counts.

ARR and baseline activity were not part of OSPI. Country‐specific approval dates came from a public sponsor dossier hosted by WHO covering 103 countries [25], with major‐market dates cross‐checked against regulatory sources. Country weights and date coverage are detailed in Table S4.

The primary OSPI used enrollment midpoint and a 5‐year horizon: country maturity was 0 before approval, increased linearly for 5 years, and remained 1 thereafter. Countries without verified dates contributed 0; a known‐date analysis renormalized available weights. OSPI represents regulatory‐environment maturity, not launch, reimbursement, prescribing, affordability, or patient‐level access. A worked calculation is provided in Appendix S1.

Sensitivity analyses varied maturation horizon, recruitment timing, approval and date assumptions, and Scandinavian off‐label rituximab. Geographic checks reweighted or deleted countries and simulated unknown arm‐by‐country allocation while preserving randomized totals. Program‐set analyses excluded FENhance, GEMINI plus FENhance, or all three BTK inhibitor programs (Appendix S1 and Table S8).

### 2.4 Statistical Analysis

Analyses were descriptive and exploratory. Scatterplots and Spearman correlations related reference‐arm ARR to enrollment midpoint and prior‐year relapse activity; secondary analyses evaluated Gd + positivity, Gd + lesion count, T2 lesion volume, alternative time scales, and program‐level aggregation. Unweighted ordinary least‐squares models estimated ARR difference per 10 OSPI points with 95% confidence intervals and *R* ^2^. Associations were not interpreted causally.

Arm‐level analyses included 15 treated‐reference arms. Recruitment timing, baseline characteristics, and OSPI pooled sister trials by randomized reference‐arm sample size; ARR used ARR‐analysis sample‐size weights. Program‐level prior‐year relapse means were rounded to one decimal before ranking. For the time‐adjusted stress test, ranked OSPI and ARR were separately regressed on ranked recruitment midpoint, and the residuals were correlated using Pearson correlation. Disability outcomes used the approximately 2‐year program series; crude proportions and preliminary pooled FENhance CDP12 were sensitivities. Other sensitivities used modern teriflunomide programs or exploratory program‐set exclusions.

Spearman coefficients used 95% bias‐corrected and accelerated bootstrap confidence intervals from 50,000 resamples. Programs were resampled as clusters for arm analyses and directly for program analyses; percentile intervals were sensitivity analyses. Constant‐rank resamples were excluded, and intervals were not multiplicity adjusted (Table S5).

Nonindependent within‐program subgroup contrasts were summarized without pooling or inclusion in trial‐level correlations; figures were limited to MRI inflammatory activity or composite highly active disease definitions.

Analyses used Python 3.12.13, pandas 3.0.3, NumPy 2.5.0, and Matplotlib 3.11.0. *p*‐values were not used for confirmatory inference.

### 2.5 Standard Protocol Approvals, Registrations, and Patient Consents

Ethics approval and informed consent were not required because only aggregate data from published or publicly accessible sources were used; no individual participant data were analyzed.

## 3 Results

### 3.1 Reference‐Arm Event Rates Across Recruitment Eras

The analysis included 15 treated‐reference arms: 2 teriflunomide 14‐mg arms from TEMSO and TOWER, 2 interferon beta‐1a comparator arms from OPERA I/II, and 11 modern teriflunomide active‐comparator arms. The TEMSO and TOWER placebo‐arm ARRs of 0.539 and 0.500 are shown as historical context but were excluded from fitted summaries and correlations. Treated‐reference ARR declined from 0.370 in TEMSO and 0.320 in TOWER to 0.109 to 0.140 in recent BTK inhibitor‐era programs (Figure 1).

Across all 15 arms, reference‐arm ARR declined with enrollment midpoint (Spearman *ρ* = −0.906; 95% CI, −0.985 to −0.279). Results were similar using enrollment start, enrollment end, and publication year (*ρ* = −0.931, −0.899, and −0.910, respectively). Among 11 modern teriflunomide active‐comparator arms, *ρ* was −0.769 for enrollment midpoint and −0.795 for publication year. Program‐level aggregation yielded *ρ* = −0.929 across all 9 programs and −0.771 across the 6 modern teriflunomide programs, indicating that the temporal pattern was not created by counting sister trials separately (Table S5).

### 3.2 Baseline Activity and Event Yield

Baseline prior‐year relapse activity was the clearest publicly available correlate of reference‐arm ARR. ARR increased with baseline prior‐year relapse activity (*ρ* = 0.739; 95% CI, 0.158 to 0.928; *n* = 13; Figure 2A), while baseline relapse activity declined with enrollment midpoint (*ρ* = −0.725; 95% CI, −0.965 to −0.157; *n* = 13; Figure 2B). At program level, after preserving the one‐decimal precision of source values, the corresponding estimates were *ρ* = 0.788 and *ρ* = −0.784 (*n* = 8 for both; Table S5).

**FIGURE 2:**
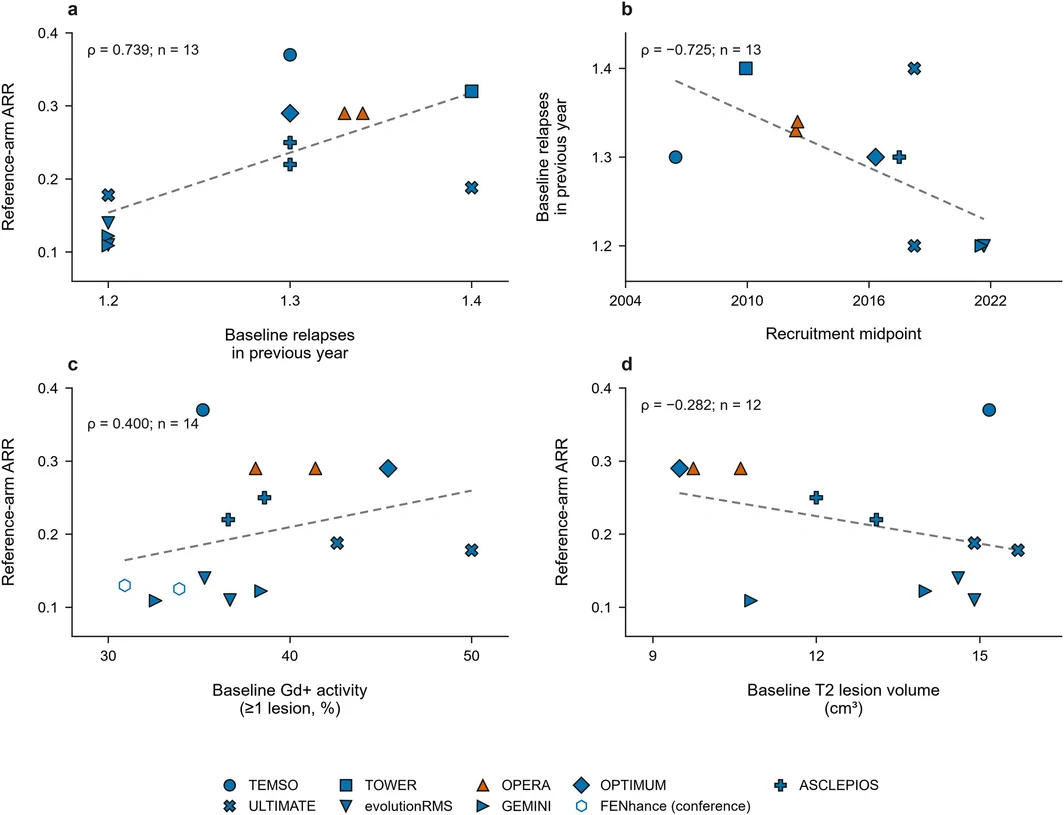
Baseline activity markers and reference‐arm ARR. (a) Relates reference‐arm ARR to baseline prior‐year relapse activity; (b) shows baseline prior‐year relapse activity over the recruitment era; (c, d) relate reference‐arm ARR to baseline Gd + positivity and T2 lesion volume, respectively. Blue markers denote teriflunomide reference arms, orange markers denote OPERA interferon beta‐1a arms, and marker shape identifies the trial program. Open hexagons denote preliminary conference data from FENhance, which contributes only to panel c because baseline prior‐year relapse activity and T2 lesion volume were not publicly reported. TEMSO and TOWER contribute their teriflunomide 14‐mg arms; TOWER is absent from the MRI panels because MRI endpoints were not collected. Dashed lines are unweighted least‐squares summaries and are descriptive only. Baseline relapse values are shown as reported and were often rounded, resulting in tied values. T2 lesion volume is shown in cm^3^; definitions differ by source. ARR, annualized relapse rate; Gd+, gadolinium‐enhancing; MRI, magnetic resonance imaging.

MRI markers were less consistently informative than relapse history. Baseline Gd + positivity was available for 14 of 15 arms and Gd + lesion count for 11 (Figure S1). Both tended to be lower in later‐enrolling arms (*ρ* = −0.445 and −0.513, respectively), but their associations with reference‐arm ARR differed (*ρ* = 0.400 [95% CI, −0.352 to 0.743; Figure 2C] and 0.566 [95% CI, −0.330 to 0.884], respectively; Table S5). These aggregate results were compatible with lower inflammatory activity but did not establish a common MRI explanation because programs reported different and incomplete baseline summaries.

T2 lesion volume was available for 12 of 15 arms (Figure S1). It did not show a monotonic explanatory relationship with reference‐arm ARR across all arms (*ρ* = −0.282; 95% CI, −0.941 to 0.583; *n* = 12; Figure 2D) or modern teriflunomide arms (*ρ* = −0.301; 95% CI, −1.000 to 0.824; *n* = 9), and it did not decline with enrollment midpoint (*ρ* = 0.347; 95% CI, −0.605 to 0.910; *n* = 12; Table S5).

Published study data provided an illustrative check that reference‐arm event yield remained higher among activity‐enriched participants. ARR was 0.280 versus 0.530 in TEMSO participants without versus with at least 1 baseline Gd + lesion, 0.250 versus 0.350 in the corresponding OPERA strata, and 0.186 versus 0.496 in the ULTIMATE non‐highly‐active versus highly active subgroups. These nonindependent contrasts differed in subgroup definition and reporting and were not pooled (Figure S2 and Table S6).

### 3.3 Disability Outcomes and Relapse Ascertainment

EDSS‐based CDP12 was reported for all nine programs and CDP24 for eight; FENhance contributed preliminary EDSS‐only CDP12 data but no CDP24 estimate (Table S10). In eight peer‐reviewed programs, selected approximately 2‐year CDP12 and CDP24 estimates declined with enrollment midpoint (*ρ* = −0.905 and −0.790). Associations attenuated with crude proportions (*ρ* = −0.429 and −0.381), and adding preliminary pooled FENhance EDSS‐only CDP12 attenuated the selected CDP12 association to *ρ* = −0.583. Disability trends were sensitive to reporting metric and effective follow‐up; crude event proportions were not directly equivalent to fixed‐time Kaplan–Meier estimates. No disability effect was pooled.

Objective relapse confirmation was documented across eras; independent central committees were verified for ULTIMATE, evolutionRMS, and GEMINI. Incomplete reporting precluded a monotonic adjudication variable (Table S11B). All nine programs had recent clinical and/or MRI activity gates (Table S11A).

### 3.4 Operational Selection Pressure Index

Verified approval dates covered 93.4% to 100.0% of each program's geography weights (Table S4). Midpoint OSPI was 0 for TEMSO, TOWER, OPERA, and OPTIMUM; 1.1 for ASCLEPIOS; 7.3 for ULTIMATE; and 65.2–80.5 for GEMINI, evolutionRMS, and FENhance (Figure 3A and Table S7). Thus, six programs scored 0–7.3 and three BTK inhibitor‐era programs scored 65.2–80.5, primarily contrasting eras rather than a dense continuum.

**FIGURE 3:**
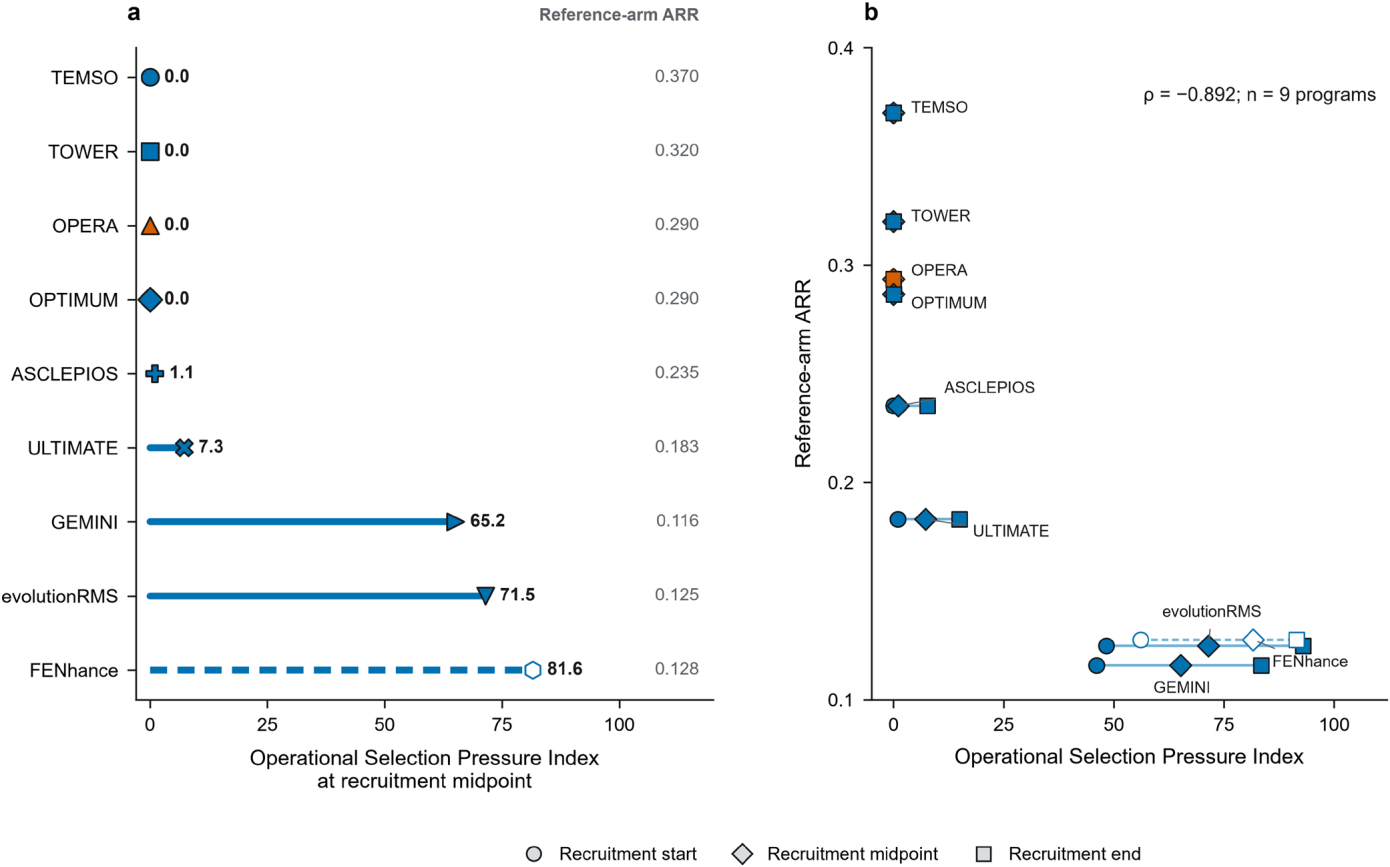
Operational selection pressure in RMS trials. (a) displays the geography‐weighted maturity of the anti‐CD20 treatment ecosystem at recruitment midpoint, with reference‐arm ARR shown for orientation but not included in score calculation. For each recruiting country, maturity increases linearly from 0 at ocrelizumab approval to 1 after 5 years; program OSPI is 100 times the participant‐ or site‐weighted mean. In the observed data, six program scores were 0–7.3 and three BTK inhibitor‐era scores were 65.2–80.5, so the display primarily contrasts eras rather than a densely sampled continuum. (b) plots ARR against OSPI under recruitment start, midpoint, and end scenarios; fixed‐size marker shapes denote scenario timing, and lines connect scenarios within each program. The coincident OPERA and OPTIMUM trajectories are minimally displaced vertically for visibility. Blue denotes teriflunomide reference arms and orange denotes OPERA interferon beta‐1a arms. FENhance is shown with a dashed line and open markers because its ARR is based on preliminary conference data. OSPI is an ecological regulatory‐availability proxy and does not measure patient‐level access or selection or an effect independent of calendar time. ARR, annualized relapse rate; OSPI, operational selection pressure index; RMS, relapsing multiple sclerosis.

Across 9 programs, higher midpoint OSPI was associated with lower ARR (*ρ* = −0.892; 95% CI, −1.000 to −0.569; Figure 3B). Excluding FENhance yielded *ρ* = −0.919 (*n* = 8); excluding GEMINI plus FENhance, −0.915 (*n* = 7); and excluding all 3 BTK inhibitor programs, −0.857 (*n* = 6), although 4 of 6 remaining scores were zero (Tables S5 and S8). Each 10‐point higher OSPI corresponded to an exploratory ARR difference of −0.0227 (95% CI, −0.0349 to −0.0104; *R* ^2^ = 0.733).

Start/end timing and 3‐ to 7‐year maturation horizons yielded *ρ* from −0.953 to −0.843. Alternative country weighting and deletion yielded *ρ* from −0.843 to −0.909, with a maximum 11.0‐point score shift. Fifty thousand simulations of unknown arm allocation retained *ρ* = −0.892 throughout the central 95% interval. Scandinavian off‐label rituximab changed scores by at most 1.85 points and yielded *ρ* = −0.902 (Table S8).

In the time‐adjusted stress test, the Pearson correlation between residualized ranks was −0.020 (Table S8), indicating negligible residual rank association after adjustment for recruitment midpoint.

## 4 Discussion

In this analysis, treated‐reference ARR and prior‐year relapse activity declined across trial eras; MRI measures offered no consistent explanation, and disability trends depended on reporting metric. OSPI made recruitment geography and anti‐CD20 approval timing explicit, but scores were concentrated in three BTK inhibitor‐era programs. These ecological findings describe correlated population and treatment‐context changes; they do not establish comparator avoidance or a single mechanism.

These findings extend rather than replicate placebo‐era literature. Earlier reviews documented declining placebo‐arm relapse rates and related them to baseline activity, eligibility, study duration, and regression to the mean [1, 4, 6]. Predictive work similarly showed that trial interpretation depends on the randomized population's baseline risk [7]. The decline in treated‐reference ARR became conspicuous when evolutionRMS placed its teriflunomide results beside earlier programs [13]. We instead asked whether recruitment timing, baseline activity, and country‐specific treatment context could help explain and potentially anticipate event yield in active‐comparator trials. Our contribution is to relate declining treated‐reference event yield to reported baseline activity and to operationalize the timing and geography of a hypothesized recruitment context.

Baseline relapse history provided the clearest link between recruited case mix and event yield. Its decline supports less clinically active later populations, although rounded aggregate means cannot identify individual selection. Within‐program contrasts provided a plausibility check: ARR was higher with baseline Gd + activity in TEMSO and OPERA and with highly active disease in ULTIMATE (Figure S2 and Table S6). Definitions differed and estimates were nonindependent, precluding pooling, but their consistent direction indicates that activity‐enriched participants still generated appreciably more events. The unresolved question is how often such patients remained available to later programs.

Changing diagnostic criteria provide an alternative explanation. Successive McDonald revisions can produce stage migration and an apparently milder prognosis without changing an individual's course [26]. In a prospective clinically isolated syndrome cohort, the 2017 criteria increased baseline diagnoses but reduced specificity for a second attack [27]. All nine programs imposed recent clinical and/or MRI activity thresholds (Table S11A). These coarse gates constrain but do not eliminate stage migration or equalize future relapse risk.

Public MRI data could not establish whether lower inflammatory lesion burden mediated the ARR decline. MRI activity relates to relapse risk and trial‐level relapse effects [11, 12], but programs reported non‐equivalent subsets of Gd + positivity, lesion count, and T2 lesion volume (Figure S1). Binary Gd + positivity combines different degrees of enhancement, while volume might add granularity if consistently acquired and reported. Baseline Gd + T1 lesion volume was too rarely available to test this hypothesis, and our data do not show that it would outperform simpler measures.

Outcome ascertainment showed no simple era transition: objective relapse confirmation was present early, whereas independent committees were verified in several later programs (Table S11B). Selected approximately 2‐year CDP12 and CDP24 estimates declined, but the pattern weakened with crude estimators and preliminary FENhance conference data (Table S10). These comparisons address event yield, not whether DMTs modify long‐term disability or are merely symptomatic.

OSPI operationalized when and where recruitment overlapped with a prespecified anti‐CD20 transition. Ocrelizumab was chosen because its global chronology marked a clinically important step toward routinely available high‐efficacy B‐cell therapy [25]. US claims support adoption after its 2017 entry (Table S9) [28]. Clustered scores primarily separate earlier from three BTK inhibitor‐era programs. Their near‐zero time‐adjusted association shows that OSPI cannot be separated from calendar era here; it is an exposure‐context description, not an independent effect.

Country reweighting, deletion, arm‐allocation simulation, and Nordic rituximab modeling changed rankings little but could not validate country‐level outcomes because country‐by‐arm relapses and person‐time were unavailable. Investigational‐treatment risk could be another influence: GEMINI was fully enrolled when the global tolebrutinib recruitment pause was announced [29], but earlier safety perceptions remain unmeasured. Sparse program exclusions preserved a negative ordering but do not estimate a hold effect.

Anti‐CD20 availability also helped reshape RMS treatment strategy from escalation toward earlier high‐efficacy therapy. Observational cohorts later supported this transition: early intensive treatment was associated with better outcomes than delayed escalation [8], and contrasting Swedish and Danish strategies illustrated the consequences of embedding early high‐efficacy treatment, particularly rituximab, in routine care [9]. Once such treatment is a legitimate first‐line option, randomization to a platform or moderate‐efficacy comparator may become unattractive even without conventional highly active disease. Eligibility defines who could enter a trial, but not who remains available for randomization.

Analogous problems appear in other rapidly evolving fields. Control‐arm overperformance occurred in 43% of evaluable Phase 3 oncology trials [30], a renal cancer trial became infeasible after a new standard emerged [31], and 61% of contemporary cardiovascular trials overestimated control‐event rates and 82% overestimated treatment effects [32]. These reports show consequences of improving care, changing comparators, and selective populations, but did not quantify whether access to competing therapy reshaped populations before enrollment.

In RMS, a high‐efficacy alternative may divert patients most likely to relapse before randomization because they decline or are not offered a platform or moderate‐efficacy comparator. Randomization cannot correct pre‐enrollment selection, and baseline tables may not reveal it. Choosing high‐efficacy treatment may be individually sound while reducing trial event yield. The lower‐risk trial can remain internally valid; the concern is planning mismatch and transportability to higher‐risk patients.

Formal eligibility should not be equated with expected event yield. Under a prespecified rate ratio and fixed follow‐up, lower comparator ARR requires greater person‐time; consequences differ for absolute effects or other estimands [2]. Conservative assumptions and blinded sample‐size re‐estimation can reduce planning risk. Activity criteria or adaptive enrichment may not recover event yield if event‐generating patients remain unavailable. High‐efficacy comparators may better preserve equipoise, but their low expected ARR must inform design. Redirecting recruitment to settings with limited treatment access is fragile, ethically difficult, and less applicable to intended care.

The ecological design, selected programs, and rounded aggregates cannot reproduce individual decisions or establish causality. OSPI captures regulatory context, not reimbursement, prescribing, refusal, diagnostic timing, safety perception, or recruitment intensity. Its clustered distribution and collinearity with era preclude an independent effect estimate. Country‐by‐arm outcomes and person‐time were unavailable; robustness checks support numerical stability, not participant‐, country‐, or period‐level validation.

The major constraint was inconsistent public reporting. CONSORT specifies general recruitment and baseline reporting [33], but relevant disease‐specific detail was absent or dispersed. Reporting diagnostic criteria, activity gates, recruitment timing and geography, relapse ascertainment, disability estimators, and reference‐arm outcomes would enable replication and prospective validation without unrestricted participant data.

Expected relapse yield is not a stable property of a comparator; it emerges from the recruited population and treatment environment. OSPI makes one component measurable, but its mechanism requires screening and country‐ or period‐specific outcomes. Active‐comparator design must evolve with treatment practice.

## Author Contributions

**Steffen Pfeuffer:** conceptualization, data curation, formal analysis, investigation, methodology, project administration, software, visualization, writing – original draft, writing – review and editing. **Lars Masanneck:** data curation, formal analysis, validation, investigation, writing – review and editing. **Marc Pawlitzki:** writing – review and editing, formal analysis, validation. **Hans‐Peter Hartung:** conceptualization, supervision, writing – review and editing.

## Funding

The authors have nothing to report.

## Conflicts of Interest

S.P. reports honoraria for lectures and advisory board service from Alexion, argenx, Biogen, Hexal, Merck Healthcare, Novartis, Roche, and Sanofi; reimbursement of travel expenses from Alexion, Biogen, Merck Healthcare, Neuraxpharm, and Roche; and research support from Biogen, Merck Healthcare, and Novartis; L.M. reports honoraria for lectures and consulting and reimbursement of travel expenses from Alexion, argenx, BIAL, Biogen, Merck Healthcare, Neuraxpharm, Novartis, and Roche, all outside the submitted work; L.M. also reports research funding from the German Multiple Sclerosis Foundation (DMSG), the B. Braun Foundation, and the German Research Foundation (DFG); M.P. reports honoraria for lectures and reimbursement of travel expenses from Alexion, argenx, Bayer HealthCare, Biogen, Hexal, Merck Healthcare, Novartis, Roche, Sanofi, Takeda, and Teva; M.P. also reports research funding from argenx, Biogen, DEMECAN, Hexal, Horizon Therapeutics, Merck Healthcare, Novartis, Roche, Takeda, Teva, Viatris, the German Multiple Sclerosis Foundation (DMSG), the B. Braun Foundation, and the German Alzheimer Society; H.‐P.H. reports fees for steering committee service from TG Therapeutics; data monitoring committee service from Merck Healthcare and Novartis; and lectures or consulting from Aurinia Pharmaceuticals, Biogen, Boehringer Ingelheim, Bristol Myers Squibb (Celgene), Neuraxpharm, and Sanofi.

## Supporting information


**Appendix S1:** ene70781‐sup‐0001‐Supinfo.docx.
**Table S1:** Reference‐arm outcomes and baseline inflammatory activity.
**Table S2:** Other baseline characteristics in the reference arms.
**Table S3:** Arm‐level source locators.
**Table S4:** Geographic recruitment weights and approval‐date provenance.
**Table S5:** Program‐clustered bootstrap confidence intervals for trial‐ and program‐level associations.
**Table S6:** Within‐program reference‐arm event yield across published baseline strata.
**Table S7:** Program‐level operational selection pressure index results.
**Table S8:** OSPI definition, timing, and data‐availability sensitivities.
**Table S9:** Regulatory, launch, and adoption context for the OSPI reference therapy.
**Table S10:** Definitions, reporting metrics, and results for 12‐ and 24‐week EDSS‐confirmed disability outcomes.
**Table S11:** Diagnostic criteria, recent activity gates, and relapse‐confirmation procedures.
**Figure S1:** Public baseline activity reporting completeness.
**Figure S2:** Reference‐arm event yield within published activity strata.

## Data Availability

All aggregate trial‐level data extracted for this study, source locators, analysis documentation, analysis code, and figure‐generation scripts will be made publicly available without restriction in a public repository upon publication. Before public release, these materials are available from the corresponding author (steffen.pfeuffer@neuro.med.uni-giessen.de) on reasonable request. No individual participant data were used.
