# Supplementary material for: Comparator Event Rates, Baseline Activity, and Operational Selection Pressure in Relapsing Multiple Sclerosis Trials: A Meta‐Epidemiological Analysis
Source: Eur J Neurol. 2026 Oct 10;33(10):e70781. doi: 10.1111/ene.70781 (PMC13653506; doi:10.1111/ene.70781)
Supplement: Supplementary file 1 [file ENE-33-e70781-s001.docx]

**Supporting Information**

**Comparator Event Rates, Baseline Activity, and Operational Selection Pressure in Relapsing Multiple Sclerosis Trials**

**Contents**

Appendix S1. Supplementary Methods	2

Table S1. Reference-arm outcomes and baseline inflammatory activity	5

Table S2. Other baseline characteristics in the reference arms	6

Table S3. Arm-level source locators	7

Table S4. Geographic recruitment weights and approval-date provenance	8

Table S5. Program-clustered bootstrap confidence intervals for trial- and program-level associations	9

Table S6. Within-program reference-arm event yield across published baseline strata	10

Table S7. Program-level Operational Selection Pressure Index results	11

Table S8. OSPI definition, timing, and data-availability sensitivities	12

Table S9. Regulatory, launch, and adoption context for the OSPI reference therapy	14

Table S10. Definitions, reporting metrics, and results for 12- and 24-week EDSS-confirmed disability outcomes	15

Table S11. Diagnostic criteria, recent activity gates, and relapse-confirmation procedures	17

Figure S1. Public baseline activity reporting completeness	19

Figure S2. Reference-arm event yield within published activity strata	20

References for Supporting Information	21

**Appendix S1. Supplementary Methods**

**Reporting framework and analytical design**

This purpose-built meta-epidemiological study examined aggregate reference-arm data rather than participant-level outcomes or pooled comparative treatment effects. Reporting was guided by recommendations for meta-epidemiological methodology research. ^29^ The randomized study arm was the unit for temporal and baseline-activity analyses; the trial program was the unit for the Operational Selection Pressure Index (OSPI). No protocol was prospectively registered. Structured extraction, data-dictionary, source-provenance, code, and output-check files were versioned during the project. Conventional intervention-effect risk-of-bias and publication-bias tests were not used because no treatment effect was synthesized; source provenance, missingness, and reporting completeness were documented instead (Tables S1-3 and Figure S1). Only aggregate data were analyzed; ethics approval was not required.

**Eligibility criteria, information sources, and search methods**

Eligible evidence comprised randomized phase 3 adult relapsing multiple sclerosis trials with a public aggregate annualized relapse rate (ARR) for a prespecified historical or contemporary reference arm. The set represented early teriflunomide 14-mg experience (TEMSO and TOWER), the transition into pivotal anti-CD20 trials (the interferon beta-1a arms of OPERA I/II), and modern teriflunomide 14-mg active-comparator programs (OPTIMUM, ASCLEPIOS I/II, ULTIMATE I/II, evolutionRMS 1/2, GEMINI 1/2, and FENhance 1/2): 15 treated-reference arms from nine programs. TEMSO and TOWER placebo arms provided historical visual context only and were excluded from fitted trends, correlations, and OSPI analyses. All eligible treated-reference arms within these purposefully defined program roles were included. Searches identified source reports and companion analyses for these programs rather than producing a record-level screening denominator.

**Source identification and data verification**

Sources were searched from inception through July 27, 2026 in PubMed, references and citing reports of the main trials, ClinicalTrials.gov, the European Union Clinical Trials Register, regulatory and health technology assessment reports, and public congress material. PubMed searches combined "multiple sclerosis" with the trial acronym, drug, reference treatment, or NCT number and with "annualized relapse rate", "baseline", "relapse", "MRI", "gadolinium", "T2 lesion", "subgroup", "country", or "enrollment". A complementary ClinicalTrials.gov screen used "teriflunomide" and "multiple sclerosis" and retained phase 3 interventional adult RMS records. No language restriction was applied. A reproducible PubMed strategy for ULTIMATE was: (("multiple sclerosis"[Title/Abstract] OR "Multiple Sclerosis"[MeSH Terms]) AND (ULTIMATE[Title/Abstract] OR ublituximab[Title/Abstract] OR teriflunomide[Title/Abstract] OR NCT03277261[All Fields] OR NCT03277248[All Fields]) AND ("annualized relapse rate"[Title/Abstract] OR baseline[Title/Abstract] OR relapse[Title/Abstract] OR MRI[Title/Abstract] OR gadolinium[Title/Abstract] OR "T2 lesion"[Title/Abstract] OR subgroup[Title/Abstract] OR country[Title/Abstract] OR enrollment[Title/Abstract])). The same syntax was repeated with each program's acronym, interventions, reference treatment, and NCT identifiers; no PubMed filters were applied. Protocols informed eligibility or acquisition methods but not observed baseline values. Main reports and supplements were preferred, followed by posted registry results, regulatory or health technology assessment documents, and public congress sources. Locators are listed in Table S3. ^1,2^

**Data collection and source reconciliation**

SP screened source records and entered data into a structured arm-level form; SP and LM verified the final evidence set and analysis fields against the cited documents. Discrepancies were resolved by source reinspection and discussion, and no machine-extracted value was accepted without human verification. No inter-reviewer agreement statistic was calculated because program and arm eligibility followed objective named-program and treatment criteria rather than a subjective classification; all final inclusions and extracted values were jointly verified. Congress or secondary values were identified by source type rather than assigned a subjective evidence score. FENhance ARR confidence limits were approximate visual readouts from preliminary conference data presented at AAN 2026. The evolutionRMS T2 lesion volume unit was recorded as cm³ after clarification that the published cm² label was typographical; numerical values were unchanged.

**Data items, MRI definitions, and missing data**

Fields sought for each arm were trial/program, publication year, recruitment start and end, treatment, randomized and ARR-analysis sample sizes, on-study ARR and 95% confidence interval, prior-year relapse activity, relapse history in the previous 2 years, age, sex, Expanded Disability Status Scale score, disease duration, prior disease-modifying therapy exposure or treatment-naive proportion, formal relapse and MRI eligibility criteria, and EDSS-based 12- and 24-week confirmed disability progression or worsening. Time since first symptoms was preferred when both symptom- and diagnosis-based durations were reported; otherwise the source definition was retained.

**MRI extraction rules**

Baseline MRI fields were gadolinium-enhancing (Gd+) T1 lesion count, proportion with at least one Gd+ T1 lesion, Gd+ T1 lesion volume, T2 lesion volume, T2 lesion count, anatomical coverage, and acquisition details. Gd+ positivity was used when directly reported or explicitly calculable as one minus the proportion without enhancing lesions. Counts, volumes, units, coverage, and dispersion were retained as reported. Baseline burden was separated from change from baseline, on-treatment new/enlarging lesions, and cumulative follow-up counts. Gd+ T1 volume and T2 count were not analyzed when no sufficient arm-level series existed. Unreported values were NR and were neither inferred nor imputed; TOWER MRI fields were not collected because MRI was not a study endpoint. Correlations used pairwise complete data.

**Recruitment timing and geographic weights**

Publication-reported first and last randomization/enrollment dates were available for 10 of 15 arms. For OPTIMUM, evolutionRMS 1/2, and FENhance 1/2, study start and the first registry transition to active, not recruiting were proxies for the unavailable last-randomization date; study-completion dates were not used. Dates were converted to fractional years, assigning month-only dates to the 15th day, and the arithmetic mean of start and end defined midpoint. Midpoint summarized time without assuming linear enrollment; start/end analyses bounded within-window uncertainty.

**Geographic recruitment data**

Country names were harmonized before regulatory linkage. Public country participant counts were used where available. European Union Clinical Trials Register counts represented total trial enrollment and were not arm-specific; GEMINI used randomized country counts from its supplement. TEMSO, FENhance 1, and FENhance 2 used trial-specific ClinicalTrials.gov country site counts because public country participant counts were unavailable. These distributions described whole-trial recruitment context, not treatment-arm allocation. Sister trials remained separate in arm-level analyses and were pooled at program level with randomized reference-arm sample-size weights. Source, data type, total weight, and approval-date coverage are reported in Table S4.

**Within-program event-yield contrasts**

Main and companion reports were searched for absolute reference-arm ARRs in mutually exclusive baseline subgroups defined by MRI activity, relapse history, Expanded Disability Status Scale score, or prior treatment. Nonindependent within-program estimates were described without adding them as trial arms, pooling across programs, or including them in trial-level correlations. Figure S2 was restricted to baseline Gd+ activity or a composite highly active disease definition; all contrasts and limitations are listed in Table S6. Targeted source updates and reviewer-requested audits were performed through September 23, 2026, without changing the included trial-program set.

**Disability outcome crosswalk**

EDSS-based CDP12 and CDP24 were retained separately from composite disability endpoints. The principal descriptive series preferred reference-arm Kaplan-Meier estimates at weeks 96-108 or month 24; a fixed-duration event proportion was used for ULTIMATE because no fixed-time estimate was reported. Crude event proportions were retained as a reporting-metric sensitivity. Preliminary FENhance EDSS-only CDP12 proportions were pooled by randomized sample size only for a sensitivity analysis; cCDP12 was excluded because it combined EDSS, walking, and upper-limb events. Definitions, estimates, and source locators are reported in Table S10.

**Operational Selection Pressure Index**

OSPI was a rule-based ecological measure of anti-CD20 treatment-environment maturity across each program's recruiting countries. Ocrelizumab was selected because country-specific regulatory chronology was publicly available across the multinational geographies and its introduction marked a clinically important transition toward B-cell-depleting high-efficacy therapy. Approval dates came from a public sponsor dossier hosted by the World Health Organization and were cross-checked against major-market regulators. ^24-26^ The dossier contained 103 country entries. Three dates preceding first global ocrelizumab approval were retained raw but treated as unavailable primarily.

**OSPI calculation and sensitivity definitions**

For country c, program t, recruitment scenario s, and maturation horizon H, maturity was m(c,t,s;H)=min[max{Y(t,s)-A(c),0}/H,1], where Y was fractional enrollment start, midpoint, or end and A was the fractional year of the exact approval date. OSPI(t,s;H)=100 x sum_c[w(c,t)m(c,t,s;H)]/sum_c[w(c,t)], where w was the country participant count or, for TEMSO and FENhance, a trial-specific ClinicalTrials.gov site-count proxy. Sister trials were pooled with randomized reference-arm sample-size weights for timing and OSPI; their reference-arm ARRs were pooled by ARR-analysis sample size. The primary midpoint, 5-year specification assigned 0 before approval, increased linearly to 1 over 5 postapproval years, and remained 1 thereafter; unknown dates contributed 0. ARR, baseline activity, and outcomes did not enter OSPI. Thus, OSPI represented regulatory-environment maturity, not launch, reimbursement, prescribing, affordability, or individual access. As an illustrative calculation, if 60% of a program's geography weight came from a country 2 years beyond approval at midpoint and 40% from a country still preapproval, OSPI would equal 100 x [(0.60 x 2/5) + (0.40 x 0)] = 24. This example is not study data and does not represent individual treatment access.

**Sensitivity and stress tests**

Alternatives used enrollment start/end; 3- and 7-year horizons; binary approval; approval or anticipated approval within 1 year; uncapped weighted postapproval years; known-date-only renormalization; and source dates as reported. Further analyses excluded FENhance, GEMINI plus FENhance, or all BTK inhibitor programs; restricted the series to six modern teriflunomide programs; or assigned full anti-CD20 pressure from 2015 in Sweden, Norway, Denmark, and Finland. Geographic checks applied equal-country and square-root weights, capped any country at 10%, removed the largest or three largest countries within each program, and removed each country globally or within programs. Fifty thousand multivariate-hypergeometric simulations allocated public participant-count totals between randomized arms while preserving each program's reference-arm total; TEMSO and FENhance remained fixed because their geographies were represented by site-count proxies (Tables S7 and S8).

**Geographic robustness and outcome feasibility**

Country-level comparator ARRs were not calculated because public sources did not provide country-by-arm relapse counts or person-time. Whole-trial country totals cannot recover either quantity. Feasibility was examined descriptively by applying each program's randomized reference-arm fraction and ARR to country totals under 1.5- and 2-year complete-follow-up scenarios; these expected counts were not treated as observed outcomes. Public regional estimates from TEMSO, OPERA, and evolutionRMS were reviewed as nonharmonized boundary checks and were not pooled.

**Statistical analysis**

Arm-level analyses used 15 treated-reference arms. Spearman correlations were Pearson correlations of average ranks and used pairwise complete observations; contributing n accompanied each coefficient. Dashed scatterplot lines were unweighted ordinary least-squares visual summaries. For the time-adjusted stress test, ranked OSPI and ARR were separately regressed on ranked recruitment midpoint, and the residuals were correlated using Pearson correlation. Program-level recruitment timing, baseline characteristics, and OSPI pooled sister trials by randomized reference-arm sample size; reference-arm ARR pooled them by ARR-analysis sample size. Program-level prior-year relapse means were rounded back to one decimal before ranking, preventing rank separation from precision absent in source reports. The analytic sample comprised all eligible arms in the defined evidence set; no a priori sample-size calculation was performed. Disability associations used the selected approximately 2-year reference-arm series; crude event proportions and preliminary pooled FENhance EDSS-only CDP12 were sensitivity analyses. No disability treatment effects were pooled.

**Bootstrap confidence intervals**

Spearman coefficients were recalculated from average ranks, with exact ties assigned average rank, using pairwise complete observations fixed before resampling. Bias-corrected and accelerated 95% confidence intervals used 50,000 deterministic bootstrap resamples. Arm-level analyses resampled trial programs with replacement as clusters and retained all eligible arms from each sampled program; program-level analyses resampled program rows. Bias correction used the bootstrap distribution relative to the observed coefficient, and acceleration used a leave-one-program-out jackknife. Resamples yielding a constant ranked variable were excluded. Percentile intervals from the 2.5th and 97.5th percentiles were calculated as a sensitivity analysis. The base random-number seed was 20260731 and was incremented deterministically by analysis. Valid replicate counts and both interval estimates are reported in Table S5.

**Regression and time-adjusted stress test**

The exploratory unweighted regression modeled program-level ARR against OSPI/10, so its slope was ARR difference per 10 OSPI points. Two-sided 95% confidence intervals used the t distribution with n-2 degrees of freedom; fit was R2. For the time-adjusted stress test, OSPI, ARR, and midpoint were ranked; ranked OSPI and ARR were separately regressed on ranked midpoint, and the Pearson correlation of residuals was the partial rank correlation. No multiplicity adjustment or confirmatory P-value framework was used, and associations were not interpreted causally. Software was Python 3.12.13, pandas 3.0.3, NumPy 2.5.0, and Matplotlib 3.11.0.

**Table S1. Reference-arm outcomes and baseline inflammatory activity**

| **Trial** | **Year** | **Enrollment** | **Reference arm** | **n randomized** | **n ARR** | **ARR (95% CI)** | **Prior-year relapses** | **Gd+ count, mean** | **>=1 Gd+, %** | **T2 lesion volume, cm3** |
| --- | --- | --- | --- | --- | --- | --- | --- | --- | --- | --- |
| TEMSO | 2011 | 2004-09-24 to 2008-03-13 | Teriflunomide 14 mg | 359 | 358 | 0.370 (0.31 to 0.44) | 1.3 | 1.81 | 35.2 | 15.17 |
| TOWER | 2014 | 2008-09-17 to 2011-02-17 | Teriflunomide 14 mg | 372 | 370 | 0.320 (0.27 to 0.38) | 1.4 | NC | NC | NC |
| OPERA I | 2017 | 2011-08-31 to 2013-02-14 | Interferon beta-1a 44 mcg SC | 411 | 411 | 0.290 (0.24 to 0.36) | 1.33 | 1.87 | 38.1 | 9.74 |
| OPERA II | 2017 | 2011-09-20 to 2013-03-28 | Interferon beta-1a 44 mcg SC | 418 | 418 | 0.290 (0.23 to 0.36) | 1.34 | 1.95 | 41.4 | 10.61 |
| OPTIMUM | 2021 | 2015-06-04 to 2017-03-30 | Teriflunomide 14 mg | 566 | 566 | 0.290 (0.254 to 0.331) | 1.3 | NR | 45.4 | 9.49 |
| ASCLEPIOS I | 2020 | 2016-10 to 2018-03 | Teriflunomide 14 mg | 462 | 462 | 0.220 (0.18 to 0.26) | 1.3 | 1.2 | 36.6 | 13.10 |
| ASCLEPIOS II | 2020 | 2016-10 to 2018-03 | Teriflunomide 14 mg | 474 | 474 | 0.250 (0.21 to 0.30) | 1.3 | 1.5 | 38.6 | 12.00 |
| ULTIMATE I | 2022 | 2017-09-22 to 2018-10-04 | Teriflunomide 14 mg | 275 | 274 | 0.188 (0.12 to 0.28) | 1.4 | 1.6 | 42.6 | 14.90 |
| ULTIMATE II | 2022 | 2017-09-22 to 2018-10-04 | Teriflunomide 14 mg | 273 | 272 | 0.178 (0.11 to 0.29) | 1.2 | 2.5 | 50.0 | 15.70 |
| evolutionRMS 1 | 2024 | 2020-06-12 to 2022-11-07 | Teriflunomide 14 mg | 564 | 564 | 0.140 (0.11 to 0.18) | 1.2 | 1.3 | 35.3 | 14.60 |
| evolutionRMS 2 | 2024 | 2020-07-02 to 2022-11-07 | Teriflunomide 14 mg | 583 | 583 | 0.110 (0.09 to 0.13) | 1.2 | 1.5 | 36.7 | 14.90 |
| GEMINI 1 | 2025 | 2020-06-25 to 2022-08-08 | Teriflunomide 14 mg | 488 | 488 | 0.122 (0.10 to 0.15) | 1.2 | 1.5 | 38.4 | 14.00 |
| GEMINI 2 | 2025 | 2020-06-25 to 2022-08-08 | Teriflunomide 14 mg | 452 | 452 | 0.109 (0.09 to 0.13) | 1.2 | 1.0 | 32.6 | 10.80 |
| FENhance 1 | 2026 | 2021-03-17 to 2024-04-10 | Teriflunomide 14 mg | 372 | 372 | 0.125 (approx. 0.097-0.152) | NR | NR | 33.9 | NR |
| FENhance 2 | 2026 | 2021-03-24 to 2023-11-17 | Teriflunomide 14 mg | 375 | 375 | 0.130 (approx. 0.102-0.166) | NR | NR | 30.9 | NR |

Abbreviations: ARR, annualized relapse rate; CI, confidence interval; Gd+, gadolinium-enhancing. NC indicates not collected; NR, not reported. FENhance CIs are approximate visual readouts from preliminary conference data presented at AAN 2026.

**Table S2. Other baseline characteristics in the reference arms**

| **Trial** | **Age, mean** | **Female, %** | **EDSS, source-reported** | **Years since symptoms** | **Years since diagnosis** | **Prior DMT or treatment-naive measure** |
| --- | --- | --- | --- | --- | --- | --- |
| TEMSO | 37.8 | 71.0 | 2.67 | 8.7 | 5.59 | Use of DMT in previous 2 years: 28.4%; no DMT in previous 2 years: 71.6% |
| TOWER | 38.2 | 69.4 | 2.71 | NR | 5.27 | Use of MS medication in previous 2 years: 126/372 (34%); no MS medication in previous 2 years: 246/372 (66%) |
| OPERA I | 36.9 | 66.2 | 2.71 | NR | 3.71 | Previous disease-modifying therapy: 117/409 (28.6%); no previous DMT: 292/409 (71.4%) |
| OPERA II | 37.4 | 67.0 | 2.79 | NR | 4.13 | Previous disease-modifying therapy: 103/417 (24.7%); no previous DMT: 314/417 (75.3%) |
| OPTIMUM | 36.8 | 65.7 | 2.56 | 7.65 | 4.8 | Any prior MS DMT: 245/566 (43.3%); prior DMT within 2 years: 211/566 (37.3%) |
| ASCLEPIOS I | 37.8 | 68.6 | 2.9 | 8.2 | 5.6 | Any prior MS DMT: 280/462 (60.6%) |
| ASCLEPIOS II | 38.2 | 67.3 | 2.9 | 8.2 | 5.5 | Any prior MS DMT: 293/474 (61.8%) |
| ULTIMATE I | 37.0 | 65.3 | 2.89 | 6.8 | 4.5 | No previous DMT in prior 5 years: 59.1%; FDA 'received appropriate MS treatment': 82/274 (29.9%) |
| ULTIMATE II | 36.2 | 64.7 | 2.96 | 7.4 | 5.0 | No previous DMT in prior 5 years: 57.0%; FDA 'received appropriate MS treatment': 87/272 (32.0%) |
| evolutionRMS 1 | 38.0 | 66.3 | 2.7 | 7.1 | NR | Naive 352/564 (62.4%); experienced 212/564 (37.6%) |
| evolutionRMS 2 | 37.0 | 63.5 | 2.7 | 6.6 | NR | Naive 385/583 (66.0%); experienced 198/583 (34.0%) |
| GEMINI 1 | 36.6 | 66.6 | 2.4 | NR | 4.6 | Previous DMT: 197/488 (40.4%) |
| GEMINI 2 | 36.1 | 64.8 | 2.3 | NR | 3.8 | Previous DMT: 152/452 (33.6%) |
| FENhance 1 | 35.8 | 68.0 | 2.5 (median) | 3.4 (median) | 1.2 (median) | Prior DMT use: 61/372 (16.4%); defined as any DMT in year before screening or anti-CD20 in 3 years before screening |
| FENhance 2 | 34.8 | 65.6 | 2.0 (median) | 2.6 (median) | 0.6 (median) | Prior DMT use: 73/375 (19.5%); defined as any DMT in year before screening or anti-CD20 in 3 years before screening |

Disease duration fields remain in the metric and time origin reported by the original source; no conversion between time since symptom onset and time since diagnosis was attempted.

**Table S3. Arm-level source locators**

| **Trial / arm** | **Source locator** |
| --- | --- |
| TEMSO / Teriflunomide 14 mg | 5, 6. O'Connor et al. Randomized trial of oral teriflunomide for relapsing multiple sclerosis. N Engl J Med 2011;365:1293-1303. DOI 10.1056/NEJMoa1014656; PMID 21991951; main article Table 1, p. 1297; supplementary appendix; O'Connor et al. Randomized trial of oral teriflunomide for relapsing multiple sclerosis. N Engl J Med 2011;365:1293-1303. DOI 10.1056/NEJMoa1014656; main article Table 2, pp. 1298-1299; Wolinsky et al. Magnetic resonance imaging outcomes from a phase III trial of teriflunomide. Mult Scler 2013;19:1310-1319. DOI 10.1177/1352458513475723; Table 1, p. 1312; recruitment window: O'Connor et al. N Engl J Med. 2011;365:1293-1303; Results text |
| TOWER / Teriflunomide 14 mg | 1, 7. ClinicalTrials.gov results, NCT00751881, baseline characteristic table; Confavreux et al. Oral teriflunomide for patients with relapsing multiple sclerosis (TOWER). Lancet Neurol 2014;13:247-256. DOI 10.1016/S1474-4422(13)70308-9; PMID 24461574; main article Table 1, p. 250, Table 2, p. 251, and text noting no MRI endpoints, p. 249; recruitment window: Confavreux et al. Lancet Neurol. 2014;13:247-256; Results text |
| OPERA I / Interferon beta-1a 44 mcg SC | 8, 9. CADTH Clinical Review Report: Ocrelizumab (Ocrevus), Table 6 / NCBI Bookshelf table cl3.table6; Hauser et al. Ocrelizumab versus Interferon Beta-1a in Relapsing Multiple Sclerosis. N Engl J Med 2017;376:221-234. DOI 10.1056/NEJMoa1601277; PMID 28002679; main article Table 1; recruitment window: Hauser et al. N Engl J Med. 2017;376:221-234; Results text |
| OPERA II / Interferon beta-1a 44 mcg SC | 8, 9. CADTH Clinical Review Report: Ocrelizumab (Ocrevus), Table 6 / NCBI Bookshelf table cl3.table6; Hauser et al. Ocrelizumab versus Interferon Beta-1a in Relapsing Multiple Sclerosis. N Engl J Med 2017;376:221-234. DOI 10.1056/NEJMoa1601277; PMID 28002679; main article Table 1; recruitment window: Hauser et al. N Engl J Med. 2017;376:221-234; Results text |
| OPTIMUM / Teriflunomide 14 mg | 1, 10-12. Kappos et al. Ponesimod Compared With Teriflunomide in Patients With Relapsing Multiple Sclerosis in the Active-Comparator Phase 3 OPTIMUM Study. JAMA Neurol 2021;78:558-567. DOI 10.1001/jamaneurol.2021.0405; main article Table 1, p. 561 and Table 2, p. 563; supplement; FDA CDER Clinical Review Ponvory (ponesimod), NDA 213498Orig1s000, Table 10, pp. 49-50; EMA Ponvory public assessment report, EMA/CHMP/206970/2021, Tables 6-9, pp. 71-73; recruitment window: ClinicalTrials.gov NCT02425644 current record and history API |
| ASCLEPIOS I / Teriflunomide 14 mg | 13, 14. Hauser et al. Ofatumumab versus Teriflunomide in Multiple Sclerosis. N Engl J Med 2020;383:546-557. DOI 10.1056/NEJMoa1917246; main article Table 1, p. 549; supplementary appendix; Hauser et al. Ofatumumab versus Teriflunomide in Multiple Sclerosis. N Engl J Med 2020;383:546-557. DOI 10.1056/NEJMoa1917246; main article Table 2, p. 550; CADTH Common Drug Review Clinical Review Report: Ofatumumab (Kesimpta), April 2021; Table 7, pp. 37-38 and Table 8, p. 39; recruitment window: Hauser et al. N Engl J Med. 2020;383:546-557; Results text |
| ASCLEPIOS II / Teriflunomide 14 mg | 13, 14. Hauser et al. Ofatumumab versus Teriflunomide in Multiple Sclerosis. N Engl J Med 2020;383:546-557. DOI 10.1056/NEJMoa1917246; main article Table 1, p. 549; supplementary appendix; Hauser et al. Ofatumumab versus Teriflunomide in Multiple Sclerosis. N Engl J Med 2020;383:546-557. DOI 10.1056/NEJMoa1917246; main article Table 2, p. 550; CADTH Common Drug Review Clinical Review Report: Ofatumumab (Kesimpta), April 2021; Table 7, pp. 37-38 and Table 8, p. 39; recruitment window: Hauser et al. N Engl J Med. 2020;383:546-557; Results text |
| ULTIMATE I / Teriflunomide 14 mg | 15, 16. Steinman et al. Ublituximab versus Teriflunomide in Relapsing Multiple Sclerosis. N Engl J Med 2022;387:704-714. DOI 10.1056/NEJMoa2201904; PMID 36001711; main article Table 1, p. 708 and Table 2, p. 710; supplementary appendix; FDA CDER Clinical Review Briumvi (ublituximab-xiiy), BLA 761238Orig1s000, Study TG1101-RMS301 Tables 8-12, pp. 44-48 and Study TG1101-RMS302 Tables 24-28, pp. 64-68; recruitment window: Steinman et al. N Engl J Med. 2022;387:704-714; Results text |
| ULTIMATE II / Teriflunomide 14 mg | 15, 16. Steinman et al. Ublituximab versus Teriflunomide in Relapsing Multiple Sclerosis. N Engl J Med 2022;387:704-714. DOI 10.1056/NEJMoa2201904; PMID 36001711; main article Table 1, p. 708 and Table 2, p. 710; supplementary appendix; FDA CDER Clinical Review Briumvi (ublituximab-xiiy), BLA 761238Orig1s000, Study TG1101-RMS301 Tables 8-12, pp. 44-48 and Study TG1101-RMS302 Tables 24-28, pp. 64-68; recruitment window: Steinman et al. N Engl J Med. 2022;387:704-714; Results text |
| evolutionRMS 1 / Teriflunomide 14 mg | 1, 17. Montalban et al. Safety and efficacy of evobrutinib in relapsing multiple sclerosis (evolutionRMS1 and evolutionRMS2). Lancet Neurol 2024. DOI 10.1016/S1474-4422(24)00328-4; PMID 39307151; main article Table 1, p. 6 and Table 2, p. 7; supplementary appendix; recruitment window: Montalban et al. Lancet Neurol. 2024;23:1119-1132; ClinicalTrials.gov NCT04338022 history API |
| evolutionRMS 2 / Teriflunomide 14 mg | 1, 17. Montalban et al. Safety and efficacy of evobrutinib in relapsing multiple sclerosis (evolutionRMS1 and evolutionRMS2). Lancet Neurol 2024. DOI 10.1016/S1474-4422(24)00328-4; PMID 39307151; main article Table 1, p. 6 and Table 2, p. 7; supplementary appendix; recruitment window: Montalban et al. Lancet Neurol. 2024;23:1119-1132; ClinicalTrials.gov NCT04338061 history API |
| GEMINI 1 / Teriflunomide 14 mg | 3. Oh et al. Tolebrutinib versus Teriflunomide in Relapsing Multiple Sclerosis. N Engl J Med 2025;392:1893-1904. DOI 10.1056/NEJMoa2415985; PMID 40202623; main article Table 1, p. 1897 and Table 2, pp. 1898-1899; supplementary appendix; recruitment window: Oh et al. N Engl J Med. 2025;392:1893-1904; Results text |
| GEMINI 2 / Teriflunomide 14 mg | 3. Oh et al. Tolebrutinib versus Teriflunomide in Relapsing Multiple Sclerosis. N Engl J Med 2025;392:1893-1904. DOI 10.1056/NEJMoa2415985; PMID 40202623; main article Table 1, p. 1897 and Table 2, pp. 1898-1899; supplementary appendix; recruitment window: Oh et al. N Engl J Med. 2025;392:1893-1904; Results text |
| FENhance 1 / Teriflunomide 14 mg | 1, 4, 18. Oh et al. Efficacy and Safety of Fenebrutinib vs Teriflunomide in Relapsing Multiple Sclerosis. Official sponsor-hosted AAN 2026 presentation, baseline slide 5 and ARR slide 6. ARR point estimates were printed numerically; 95% CIs were approximate graphical readouts. Recruitment window: ClinicalTrials.gov current record and history API. |
| FENhance 2 / Teriflunomide 14 mg | 1, 4, 18. Oh et al. Efficacy and Safety of Fenebrutinib vs Teriflunomide in Relapsing Multiple Sclerosis. Official sponsor-hosted AAN 2026 presentation, baseline slide 5 and ARR slide 6. ARR point estimates were printed numerically; 95% CIs were approximate graphical readouts. Recruitment window: ClinicalTrials.gov current record and history API. |

Each locator identifies the source used for the values in Tables S1 and S2. Regulatory-review values were used when they supplied baseline fields absent from the main publication or supplement.

**Table S4. Geographic recruitment weights and approval-date provenance**

| **Trial** | **Countries, n** | **Weight total** | **Weight type** | **Verified approval-date weight, %** | **Geography source** |
| --- | --- | --- | --- | --- | --- |
| TEMSO | 21 | 21 | CT.gov site-count proxy | 100.0 | 1. ClinicalTrials.gov NCT00134563 country-site listing; site-count proxy. |
| TOWER | 26 | 1169 | EU CTR country-level total participant count (no arm split) | 98.5 | 2. EU Clinical Trials Register, EudraCT 2007-004452-36 results. |
| OPERA I | 32 | 821 | EU CTR country-level total participant count (no arm split) | 100.0 | 2. EU Clinical Trials Register, EudraCT 2010-020337-99 results. |
| OPERA II | 24 | 835 | EU CTR country-level total participant count (no arm split) | 100.0 | 2. EU Clinical Trials Register, EudraCT 2010-020315-36 results. |
| OPTIMUM | 28 | 1133 | EU CTR country-level total participant count (no arm split) | 100.0 | 2. EU Clinical Trials Register, EudraCT 2012-000540-10 results. |
| ASCLEPIOS I | 28 | 927 | EU CTR country-level total participant count (no arm split) | 96.8 | 2. EU Clinical Trials Register, EudraCT 2015-005418-31 results. |
| ASCLEPIOS II | 30 | 955 | EU CTR country-level total participant count (no arm split) | 96.5 | 2. EU Clinical Trials Register, EudraCT 2015-005419-33 results. |
| ULTIMATE I | 9 | 549 | EU CTR country-level total participant count (no arm split) | 100.0 | 2. EU Clinical Trials Register, EudraCT 2017-000638-75 results. |
| ULTIMATE II | 8 | 545 | EU CTR country-level total participant count (no arm split) | 100.0 | 2. EU Clinical Trials Register, EudraCT 2017-000639-15 results. |
| evolutionRMS 1 | 30 | 1124 | EU CTR country-level total participant count (no arm split) | 98.1 | 2. EU Clinical Trials Register, EudraCT 2019-004972-20 results. |
| evolutionRMS 2 | 32 | 1166 | EU CTR country-level total participant count (no arm split) | 97.3 | 2. EU Clinical Trials Register, EudraCT 2019-004980-36 results. |
| GEMINI 1 | 24 | 974 | Trial-specific randomized participant count | 97.3 | 3. Oh et al, NEJM 2025 supplementary appendix, Table S1. |
| GEMINI 2 | 25 | 899 | Trial-specific randomized participant count | 95.3 | 3. Oh et al, NEJM 2025 supplementary appendix, Table S1. |
| FENhance 1 | 23 | 158 | ClinicalTrials.gov site-count proxy | 98.1 | 1. ClinicalTrials.gov NCT04586010 location module; site-count proxy, accessed September 23, 2026. |
| FENhance 2 | 17 | 106 | ClinicalTrials.gov site-count proxy | 88.7 | 1. ClinicalTrials.gov NCT04586023 location module; site-count proxy, accessed September 23, 2026. |

Country participant counts from EU Clinical Trials Register results are total trial counts and are not arm-specific. Verified approval-date weight is the percentage of each geography weight linked to a verified country-specific date. Ocrelizumab approval dates were obtained from the publicly accessible WHO-hosted sponsor dossier (eReference 24, Appendix C, Table 18); US and EU dates were cross-checked against independent regulatory or public manufacturer sources (References for Supporting Information 25 and 26).

Table S5. Program-clustered bootstrap confidence intervals for trial- and program-level associations

| **Association** | **Analysis set** | **Observations, n** | **Programs, n** | **Spearman ρ** | **95% BCa CI** | **95% percentile CI** |
| --- | --- | --- | --- | --- | --- | --- |
| Reference-arm ARR vs enrollment midpoint | All treated-reference arms | 15 | 9 | −0.906 | −0.985 to −0.279 | −0.993 to −0.507 |
| Reference-arm ARR vs prior-year relapse activity | All complete pairs | 13 | 8 | 0.739 | 0.158 to 0.928 | 0.184 to 0.932 |
| Prior-year relapse activity vs enrollment midpoint | All complete pairs | 13 | 8 | −0.725 | −0.965 to −0.157 | −0.950 to −0.087 |
| Reference-arm ARR vs proportion with >=1 baseline Gd+ lesion | All complete pairs | 14 | 8 | 0.400 | −0.352 to 0.743 | −0.385 to 0.738 |
| Proportion with >=1 baseline Gd+ lesion vs enrollment midpoint | All complete pairs | 14 | 8 | −0.445 | −0.805 to 0.316 | −0.765 to 0.439 |
| Reference-arm ARR vs baseline Gd+ lesion count | All complete pairs | 11 | 6 | 0.566 | −0.330 to 0.884 | −0.330 to 0.884 |
| Baseline Gd+ lesion count vs enrollment midpoint | All complete pairs | 11 | 6 | −0.513 | −0.823 to 0.125 | −0.782 to 0.283 |
| Reference-arm ARR vs baseline T2 lesion volume | All complete pairs | 12 | 7 | −0.282 | −0.941 to 0.583 | −0.955 to 0.569 |
| Baseline T2 lesion volume vs enrollment midpoint | All complete pairs | 12 | 7 | 0.347 | −0.605 to 0.910 | −0.521 to 0.934 |
| Reference-arm ARR vs enrollment midpoint | Modern teriflunomide programs | 11 | 6 | −0.769 | −0.975 to 0.435 | −0.994 to 0.038 |
| Reference-arm ARR vs baseline T2 lesion volume | Modern teriflunomide programs; complete pairs | 9 | 5 | −0.301 | −1.000 to 0.824 | −1.000 to 0.652 |
| Program-level ARR vs enrollment midpoint | All programs | 9 | 9 | −0.929 | −1.000 to −0.143 | −1.000 to −0.478 |
| Program-level ARR vs enrollment midpoint | Modern teriflunomide programs | 6 | 6 | −0.771 | −1.000 to 1.000 | −1.000 to 0.091 |
| Program-level ARR vs prior-year relapse activity | All complete pairs | 8 | 8 | 0.788 | 0.257 to 0.974 | 0.258 to 0.979 |
| Program-level prior-year relapse activity vs enrollment midpoint | All complete pairs | 8 | 8 | −0.784 | −0.962 to −0.252 | −0.974 to −0.257 |
| Midpoint OSPI vs program-level ARR | All programs | 9 | 9 | −0.892 | −1.000 to −0.569 | −0.987 to −0.474 |
| Midpoint OSPI vs program-level ARR | Excluding FENhance | 8 | 8 | −0.919 | −1.000 to −0.733 | −1.000 to −0.615 |
| Midpoint OSPI vs program-level ARR | Modern teriflunomide programs | 6 | 6 | −0.771 | −1.000 to 1.000 | −1.000 to 0.091 |

Confidence intervals used 50,000 bootstrap resamples. Arm-level analyses resampled programs as clusters and retained all eligible sister-trial arms from each sampled program; program-level analyses resampled programs directly. Complete pairs were fixed before resampling. Across displayed analyses, 97.70% to 100% of resamples were valid. BCa indicates bias-corrected and accelerated; CI, confidence interval; Gd+, gadolinium-enhancing; OSPI, Operational Selection Pressure Index.

**Table S6. Within-program reference-arm event yield across published baseline strata**

| **Program** | **Reference arm** | **Activity or case-mix contrast** | **Lower-category ARR** | **Higher-category ARR** | **ARR ratio** | **Overall arm ARR** | **Source** | **Key limitation** |
| --- | --- | --- | --- | --- | --- | --- | --- | --- |
| TEMSO | Teriflunomide 14 mg | Baseline Gd+ T1 status | No Gd+ lesion: 0.28 | At least 1 Gd+ lesion: 0.53 | 1.89 | 0.37 | 19 | Subgroup-specific sample sizes were not reported |
| OPERA | Interferon beta-1a | Baseline Gd+ T1 status | No Gd+ lesion: 0.25 | At least 1 Gd+ lesion: 0.35 | 1.40 | 0.29 | 20 | Arm-specific subgroup confidence intervals were not reported |
| ULTIMATE | Teriflunomide 14 mg | Highly active disease definition | Non-highly-active: 0.186 | Highly active: 0.496 | 2.67 | Not shown | 21/22 | Subgroup values are unadjusted least-squares mean ARRs from the same post hoc negative-binomial GEE model; the differently modeled overall program ARR is not shown |
| TEMSO | Teriflunomide 14 mg | Relapse history in previous 2 years | At most 1 relapse: 0.17 | At least 4 relapses: 1.01 | 5.94 | 0.37 | 19 | Extreme categories are shown descriptively; subgroup-specific sample sizes were not reported |
| OPERA | Interferon beta-1a | Prior relapse count | At most 1 relapse: 0.24 | At least 2 relapses: 0.38 | 1.58 | 0.29 | 20 | Arm-specific subgroup confidence intervals were not reported |
| OPERA | Interferon beta-1a | Baseline EDSS | EDSS less than 4: 0.22 | EDSS at least 4: 0.35 | 1.59 | 0.29 | 20 | EDSS is not a pure inflammatory activity marker |
| OPTIMUM | Teriflunomide 14 mg | Treatment history | Treatment-naive: 0.265 | Previously treated: 0.369 | 1.39 | 0.29 | 23 | Poster-level data; subgroup sample sizes and arm-specific confidence intervals were not displayed |
| OPTIMUM | Teriflunomide 14 mg | Baseline EDSS | EDSS at most 3: 0.271 | EDSS greater than 3: 0.396 | 1.46 | 0.29 | 23 | Poster-level data; subgroup sample sizes and arm-specific confidence intervals were not displayed |

ARR ratios compare the displayed higher-category estimate with the lower-category estimate and were not pooled. Subgroup definitions differ across programs, observations are not independent of their overall arms, and these contrasts do not estimate a temporal or OSPI effect.

**Table S7. Program-level Operational Selection Pressure Index results**

| **Program** | **Trials** | **Reference-arm ARR** | **OSPI start** | **OSPI midpoint** | **OSPI end** | **Approval date known, %** | **Geography weight total** | **Geography basis** |
| --- | --- | --- | --- | --- | --- | --- | --- | --- |
| TEMSO | TEMSO | 0.370 | 0.0 | 0.0 | 0.0 | 100.0 | 21 | CT.gov site-count proxy |
| TOWER | TOWER | 0.320 | 0.0 | 0.0 | 0.0 | 98.5 | 1169 | EU CTR country-level total participant count (no arm split) |
| OPERA | OPERA I/II | 0.290 | 0.0 | 0.0 | 0.0 | 100.0 | 1656 | EU CTR country-level total participant count (no arm split) |
| OPTIMUM | OPTIMUM | 0.290 | 0.0 | 0.0 | 0.0 | 100.0 | 1133 | EU CTR country-level total participant count (no arm split) |
| ASCLEPIOS | ASCLEPIOS I/II | 0.235 | 0.0 | 1.1 | 7.7 | 96.7 | 1882 | EU CTR country-level total participant count (no arm split) |
| ULTIMATE | ULTIMATE I/II | 0.183 | 1.0 | 7.3 | 15.0 | 100.0 | 1094 | EU CTR country-level total participant count (no arm split) |
| GEMINI | GEMINI 1/2 | 0.116 | 46.1 | 65.2 | 83.5 | 96.4 | 1873 | Trial-specific randomized participant count |
| evolutionRMS | evolutionRMS 1/2 | 0.125 | 48.3 | 71.5 | 93.0 | 97.7 | 2290 | EU CTR country-level total participant count (no arm split) |
| FENhance | FENhance 1/2 | 0.128 | 56.8 | 80.5 | 87.8 | 93.4 | 264 | CT.gov site-count proxy |

Program-level timing and OSPI were pooled using randomized reference-arm sample sizes; reference-arm ARR was pooled using ARR-analysis sample sizes. The primary OSPI uses a 5-year postapproval maturity horizon. Unknown approval dates contribute 0.

**Table S8. OSPI definition, timing, and data-availability sensitivities**

| **Analysis** | **Timing** | **Maturity cap, y** | **Programs, n** | **Spearman rho** | **Maximum score shift** | **Definition** |
| --- | --- | --- | --- | --- | --- | --- |
| 3-year maturity cap; start | start | 3 | 9 | -0.843 | NA | Geography-weighted capped years since ocrelizumab approval |
| 3-year maturity cap; midpoint | midpoint | 3 | 9 | -0.944 | NA | Geography-weighted capped years since ocrelizumab approval |
| 3-year maturity cap; end | end | 3 | 9 | -0.953 | NA | Geography-weighted capped years since ocrelizumab approval |
| 5-year maturity cap; start | start | 5 | 9 | -0.843 | NA | Geography-weighted capped years since ocrelizumab approval |
| 5-year maturity cap; midpoint | midpoint | 5 | 9 | -0.892 | NA | Geography-weighted capped years since ocrelizumab approval |
| 5-year maturity cap; end | end | 5 | 9 | -0.919 | NA | Geography-weighted capped years since ocrelizumab approval |
| 7-year maturity cap; start | start | 7 | 9 | -0.843 | NA | Geography-weighted capped years since ocrelizumab approval |
| 7-year maturity cap; midpoint | midpoint | 7 | 9 | -0.892 | NA | Geography-weighted capped years since ocrelizumab approval |
| 7-year maturity cap; end | end | 7 | 9 | -0.902 | NA | Geography-weighted capped years since ocrelizumab approval |
| Modern teriflunomide-program subset | midpoint | 5 | 6 | -0.771 | NA | Exploratory analysis restricted to modern teriflunomide programs |
| 8-program sensitivity analysis excluding FENhance | midpoint | 5 | 8 | -0.919 | NA | 8-program sensitivity analysis excluding FENhance |
| Formal approval coverage only | midpoint | NA | 9 | -0.944 | NA | Binary geography-weighted approval coverage |
| Approval or within 1 year | midpoint | NA | 9 | -0.749 | NA | Binary geography-weighted approval/anticipation coverage |
| Uncapped weighted access-years | midpoint | NA | 9 | -0.892 | NA | Geography-weighted years since approval without cap |
| Known approval dates only | midpoint | 5 | 9 | -0.892 | NA | Primary 5-year index renormalized within countries with verified public approval dates |
| Source dates as reported | midpoint | 5 | 9 | -0.892 | 0.35 | Sensitivity retaining three source dates before the first global ocrelizumab approval |
| Including rituximab off-label | midpoint | 5 | 9 | -0.902 | 1.85 | Primary 5-year index with full pressure assigned in Sweden, Norway, Denmark, and Finland from 2015 |
| Partial rho controlling enrollment midpoint | midpoint-adjusted | 5 | 9 | -0.020 | NA | Rank residual association in the 9-program analysis including FENhance |
| Partial rho controlling enrollment midpoint; exclude FENhance | midpoint-adjusted | 5 | 8 | -0.098 | NA | Rank residual association in the 8-program sensitivity analysis excluding FENhance |

The partial rank correlation is a time-adjusted descriptive analysis and does not estimate a causal treatment-availability effect. Program exclusions are exploratory stress tests: after exclusion of all BTK inhibitor programs, four of six OSPI scores were tied at zero and 91.3% of bootstrap draws yielded a defined coefficient. Geographic reweighting and deletion assess numerical dependence of the score, not country-level outcome validity. The arm-allocation result is the median with central 95% simulation interval.

**Table S8 (continued). Geographic and program-set robustness analyses**

| **Analysis** | **Timing** | **Maturity cap, y** | **Programs, n** | **Spearman rho** | **Maximum score shift** | **Definition** |
| --- | --- | --- | --- | --- | --- | --- |
| Exploratory exclusion: GEMINI and FENhance | midpoint | 5 | 7 | -0.915 | NA | Exploratory program-set exclusion of GEMINI and FENhance |
| Exploratory exclusion: all BTKi programs | midpoint | 5 | 6 | -0.857 | NA | Exploratory program-set exclusion of GEMINI, evolutionRMS, and FENhance |
| Equal-country weighting | midpoint | 5 | 9 | -0.909 | 10.95 | Every recruiting country receives equal weight within each trial |
| Square-root country weighting | midpoint | 5 | 9 | -0.892 | 4.58 | Square root of the observed country geographic weight (participant or site count). |
| Country weight capped at 10% | midpoint | 5 | 9 | -0.892 | 1.82 | Country count capped at 10% of the trial geography total |
| Largest country removed | midpoint | 5 | 9 | -0.892 | 3.31 | Largest recruiting country removed within each trial |
| Three largest countries removed | midpoint | 5 | 9 | -0.843 | 4.81 | Three largest recruiting countries removed within each trial |
| Global single-country deletion | midpoint | 5 | 9 | -0.892 to -0.843 | 4.82 | Each country removed in turn from every program recruiting there |
| Unknown arm allocation simulation | midpoint | 5 | 9 | -0.892 (-0.892 to -0.892) | NA | Median (central 95% simulation interval) across 50,000 randomization-consistent allocations; site-proxy programs fixed |

Program exclusions and geographic perturbations are exploratory stress tests. Site-count proxy programs were held fixed in the arm-allocation simulation.

**Table S9. Regulatory, launch, and adoption context for the OSPI reference therapy**

| **Evidence domain** | **Observation** | **Role in the analysis** | **Source** |
| --- | --- | --- | --- |
| Country-specific regulatory chronology | Approval dates for 103 countries were available in a publicly accessible ocrelizumab application dossier hosted by WHO (Appendix C, Table 18). | Direct input to the country-weighted OSPI. | 24 |
| Major-market authorization | US approval: March 28, 2017; EU marketing authorization: January 8, 2018. | Confirms the timing of the transition in major recruiting markets. | 25, 26 |
| Initial commercial launch | The US approval announcement anticipated availability within 2 weeks; Roche reported launches in Europe and additional countries during Q1 2018. | Contextual evidence only; launch dates were not used to calculate OSPI. | 26, 27 |
| Observed treatment adoption | US claims data showed increasing infusion-therapy initiations after ocrelizumab was introduced in 2017, reaching 8.2% of adult DMT initiations in 2020. | Independent face-validity evidence only; utilization data were not used to calculate OSPI. | 28 |

Regulatory approval, commercial launch, reimbursement, and patient-level access are distinct. The latter three were not assumed from approval status and were not components of OSPI. The WHO-hosted application dossier is a publicly accessible source document and is not described as a WHO-validated regulatory database.

**Table S10A. Selected approximately 2-year EDSS-confirmed disability estimates**

| **Program** | **Reference arm** | **EDSS increase definition** | **Horizon and estimator** | **CDP12, %** | **CDP24, %** | **Source locator** |
| --- | --- | --- | --- | --- | --- | --- |
| TEMSO | Teriflunomide 14 mg | Increase >=1.0 point if baseline EDSS <=5.5; >=0.5 point if >5.5 | Week 108; Kaplan-Meier estimate at week 108 | 20.2 | 13.8 | CADTH/NCBI Table 16; EMA product information section 5.1, Table 1 |
| TOWER | Teriflunomide 14 mg | Increase >=1.0 point if baseline EDSS <=5.5; >=0.5 point if >5.5 | Week 108; Kaplan-Meier estimate at week 108 | 15.8 | 11.7 | CADTH/NCBI Table 16; EMA product information section 5.1, Table 1 |
| OPERA I/II | Interferon beta-1a 44 mcg SC | Increase >=1.0 point if baseline EDSS <=5.5; >=0.5 point if >5.5 | Through week 96; Kaplan-Meier estimate at week 96 | 15.2 | 12.0 | Main article Figures 1A-B and Table 2; CADTH clinical review summary table |
| OPTIMUM | Teriflunomide 14 mg | Increase >=1.5 points if baseline EDSS=0; >=1.0 if baseline 1.0-5.0; >=0.5 if baseline >=5.5 | Week 108; Kaplan-Meier estimate at week 108 | 13.2 | 10.5 | Main article Table 2/Figure 2D; EMA product information section 5.1, Table 3 |
| ASCLEPIOS I/II | Teriflunomide 14 mg | Increase >=1.5 points if baseline EDSS=0; >=1.0 if baseline 1-5; >=0.5 if baseline >=5.5 | Month 24; Kaplan-Meier estimate at month 24 | 15.0 | 12.0 | Main article Table 2 and Figures 1A-B; CADTH clinical review |
| ULTIMATE I/II | Teriflunomide 14 mg | Increase >=1.0 point if baseline EDSS <=5.5; >=0.5 if >5.5 | Through week 96; Article reports n (%) through a fixed 96-week trial | 5.9 | 4.8 | Main article Table 2; supplementary disability table; EMA EPAR Table 35, pp 99-100 |
| evolutionRMS 1/2 | Teriflunomide 14 mg | Increase >=1.0 point if baseline EDSS <=5.0; >=0.5 if baseline EDSS=5.5 | Week 96 Kaplan-Meier estimate; events also reported through study end; Kaplan-Meier estimate at week 96 | 9.8 | 6.5 | Main article Table 2 and Figure 3 |
| GEMINI I/II | Teriflunomide 14 mg | Increase >=1.5 points if baseline EDSS=0; >=1.0 if baseline 0.5-5.5; >=0.5 if >5.5 | Month 24; Kaplan-Meier estimate at month 24 | 11.8 | 9.3 | Main article Table 2; EMA EPAR disability section |

The descriptive series preferred fixed-time Kaplan-Meier estimates at weeks 96-108 or month 24. ULTIMATE contributed fixed-duration event proportions because fixed-time estimates were not reported. CDP indicates confirmed disability progression, worsening, or accumulation.

**Table S10B. Crude reference-arm disability event counts**

| **Program** | **Reference n** | **CDP12, n/N (%)** | **CDP24, n/N (%)** | **Horizon and source locator** |
| --- | --- | --- | --- | --- |
| TEMSO | 358 | 62/358 (17.3) | 43/358 (12.0) | Events accrued through the 108-week trial; CADTH/NCBI Table 16; EMA product information section 5.1, Table 1 |
| TOWER | 370 | 44/370 (11.9) | 33/370 (8.9) | Events accrued during the variable-duration trial; CADTH/NCBI Table 16; EMA product information section 5.1, Table 1 |
| OPERA I/II | 829 | 113/829 (13.6) | 87/829 (10.5) | Events accrued through week 96; Main article Figure 1A and Table 2; CADTH clinical review summary table |
| OPTIMUM | 566 | 70/566 (12.4) | 56/566 (9.9) | Events accrued through week 108; Main article Table 2/Figure 2D; EMA product information section 5.1, Table 3 |
| ASCLEPIOS I/II | 931 | 125/931 (13.4) | 99/931 (10.6) | Events accrued during the treatment epoch (variable follow-up up to 30 months; median 1.6 years); Main article Table 2 and Figure 1A; CADTH clinical review |
| ULTIMATE I/II | 546 | 32/546 (5.9) | 26/546 (4.8) | Events accrued through week 96; Main article Table 2; EMA EPAR Table 35, p 99 |
| evolutionRMS 1/2 | 1147 | 110/1147 (9.6) | 67/1147 (5.8) | Events accrued to the event-driven primary analysis (variable follow-up up to 156 weeks); Main article Table 2 and Figure 3 |
| GEMINI I/II | 940 | 144/940 (15.3) | 106/940 (11.3) | Events accrued during the event-driven trial (median follow-up 139 weeks); Main article Table 2; EMA EPAR disability section |
| FENhance 1 | 372 | 62/372 (16.7) | NR | Events accrued through the presented data cutoff; preliminary public conference data; no fixed-time estimate |
| FENhance 2 | 375 | 56/375 (14.9) | NR | Events accrued through the presented data cutoff; preliminary public conference data; no fixed-time estimate |

Crude proportions are a reporting-metric sensitivity. They reflect study-wide event accrual and are not directly equivalent to fixed-time Kaplan-Meier estimates; they do not account for censoring or unequal effective observation time. FENhance values are preliminary public conference data and EDSS-only CDP12 components; composite cCDP12 was excluded. NR indicates not reported.

**Table S11A. Diagnostic criteria and recent activity gates**

| **Program** | **Diagnostic criteria** | **Recent activity gate** | **MRI route** | **Source locator** |
| --- | --- | --- | --- | --- |
| TEMSO | McDonald criteria; relapsing MS | At least 2 relapses in prior 2 years or at least 1 in prior year | No | FDA Aubagio medical review p 63; TEMSO main report and supplement |
| TOWER | McDonald criteria; relapsing MS | At least 2 relapses in prior 2 years or at least 1 in prior year | No | TOWER publication Methods/Procedures |
| OPERA I/II | 2010 McDonald criteria; relapsing MS | At least 2 relapses in prior 2 years or at least 1 in prior year | No | CADTH Ocrevus clinical review, Outcomes/Relapse; OPERA main report |
| OPTIMUM | Relapsing MS from onset | At least 1 relapse in prior year or 2 in prior 2 years, or at least 1 Gd-enhancing lesion within 6 months | Yes | EMA Ponvory assessment pp 65-66; OPTIMUM main report |
| ASCLEPIOS I/II | 2010 McDonald criteria; RMS including active SPMS | At least 1 relapse in prior year or 2 in prior 2 years, or positive Gd-enhanced MRI in prior year | Yes | ASCLEPIOS main report; CADTH Kesimpta review |
| ULTIMATE I/II | 2010 McDonald criteria; RMS | At least 2 relapses in prior 2 years, 1 in prior year, or T1 Gd-enhancing lesion in prior year | Yes | FDA Briumvi summary review p 15; ULTIMATE II protocol Appendix E |
| evolutionRMS 1/2 | 2017 McDonald criteria; RMS | At least 1 documented relapse in prior 2 years plus either 1 in prior year or at least 1 Gd-enhancing T1 lesion within 6 months | Yes | evolutionRMS main report; evolutionRMS 1 protocol section 8.2.8 |
| GEMINI I/II | 2017 McDonald criteria; RMS | At least 1 relapse in prior year or 2 in prior 2 years, or at least 1 Gd-enhancing lesion in prior year | Yes | GEMINI main report; GEMINI II protocol relapse-assessment section |
| FENhance 1/2 | 2017 McDonald criteria; RMS | At least 2 documented clinical relapses within the previous 2 years or 1 documented clinical relapse within the previous 12 months, or at least 1 T1 Gd+ lesion on MRI within the previous 12 months | Yes | CTIS public protocols, FENhance 1 (EU Trial 2022-502609-14-00; GN41851) and FENhance 2 (EU Trial 2022-502618-95-00; GN42272), inclusion criteria. |

All nine programs had a verified explicit recent clinical and/or MRI activity threshold. Gd+ indicates gadolinium-enhancing; MRI, magnetic resonance imaging; RMS, relapsing multiple sclerosis.

**Table S11B. Objective relapse confirmation and adjudication**

| **Program** | **Objective relapse confirmation** | **Independent or central adjudication** | **Source locator** |
| --- | --- | --- | --- |
| TEMSO | Treating neurologist assessed suspected relapse; an independent certified examiner supplied EDSS and functional-system documentation | Not established | FDA Aubagio medical review p 63; TEMSO main report and supplement |
| TOWER | Treating neurologist assessed relapse; certified examining neurologist supplied EDSS at scheduled and suspected-relapse visits | Not established | TOWER publication Methods/Procedures |
| OPERA I/II | Blinded examining investigator independently assessed EDSS; protocol-relapse criteria were applied to site data | Manufacturer adjudication; not described as an independent central committee | CADTH Ocrevus clinical review, Outcomes/Relapse; OPERA main report |
| OPTIMUM | Efficacy assessor supplied EDSS/functional-system scores; treating neurologist confirmed relapse against objective thresholds | Not established | EMA Ponvory assessment pp 65-66; OPTIMUM main report |
| ASCLEPIOS I/II | Protocol-defined confirmed relapse | Not reported in reviewed public source | ASCLEPIOS main report; CADTH Kesimpta review |
| ULTIMATE I/II | Treating and examining neurologists assessed suspected events | Yes; independent relapse adjudication panel | FDA Briumvi summary review p 15; ULTIMATE II protocol Appendix E |
| evolutionRMS 1/2 | Primary ARR used adjudicated qualified relapses | Yes; blinded endpoint adjudication committee | evolutionRMS main report; evolutionRMS 1 protocol section 8.2.8 |
| GEMINI I/II | Examining investigator/rater supplied EDSS and relapse documentation | Yes; relapse adjudication committee | GEMINI main report; GEMINI II protocol relapse-assessment section |
| FENhance 1/2 | Treating investigator documented suspected relapse; examining investigator independently assessed EDSS/FSS; protocol-defined relapses were derived by the Sponsor according to prespecified criteria | No independent or central relapse-adjudication committee specified in the reviewed protocol | CTIS public protocols, FENhance 1 (GN41851) and FENhance 2 (GN42272), relapse-assessment and primary efficacy endpoint sections |

Objective confirmation and independent central adjudication were coded separately. Not established or not reported does not mean that a procedure was absent.

Green indicates reported/extractable; gray, not reported or not located; black, not collected by design. FENhance was retained on the basis of preliminary conference data; exclusion is reported as a sensitivity analysis.

Gray circles indicate lower-activity categories; green circles, activity-enriched categories; orange diamonds, the corresponding overall reference-arm estimate. Confidence intervals are shown when reported. The ULTIMATE non-highly-active estimate was available from a public conference poster.

**References for Supporting Information**

1. ClinicalTrials.gov. Study records and results tables for included relapsing multiple sclerosis trials. Accessed July 24, 2026. https://clinicaltrials.gov/.

2. European Union Clinical Trials Register. Study results for included relapsing multiple sclerosis trials. Accessed July 24, 2026. https://www.clinicaltrialsregister.eu/.

3. Oh J, Arnold DL, Cree BAC, et al. Tolebrutinib versus teriflunomide in relapsing multiple sclerosis. N Engl J Med. 2025;392:1893-1904.

4. ClinicalTrials.gov. FENhance 1 (NCT04586010) and FENhance 2 (NCT04586023) study records and history. Accessed September 23, 2026. https://clinicaltrials.gov/study/NCT04586010; https://clinicaltrials.gov/study/NCT04586023.

5. O'Connor P, Wolinsky JS, Confavreux C, et al. Randomized trial of oral teriflunomide for relapsing multiple sclerosis. N Engl J Med. 2011;365:1293-1303.

6. Wolinsky JS, Narayana PA, O'Connor P, et al. Magnetic resonance imaging outcomes from a phase III trial of teriflunomide. Mult Scler. 2013;19:1310-1319.

7. Confavreux C, O'Connor P, Comi G, et al. Oral teriflunomide for patients with relapsing multiple sclerosis (TOWER): a randomised, double-blind, placebo-controlled, phase 3 trial. Lancet Neurol. 2014;13:247-256.

8. Canadian Agency for Drugs and Technologies in Health. Clinical review report: ocrelizumab (Ocrevus). CADTH Common Drug Review, 2017. Accessed July 24, 2026. https://www.ncbi.nlm.nih.gov/books/NBK534378/.

9. Hauser SL, Bar-Or A, Comi G, et al. Ocrelizumab versus interferon beta-1a in relapsing multiple sclerosis. N Engl J Med. 2017;376:221-234.

10. Kappos L, Fox RJ, Burcklen M, et al. Ponesimod compared with teriflunomide in patients with relapsing multiple sclerosis in the active-comparator phase 3 OPTIMUM study: a randomized clinical trial. JAMA Neurol. 2021;78:558-567.

11. US Food and Drug Administration. Clinical review: Ponvory (ponesimod), NDA 213498. 2021. Accessed July 24, 2026. https://www.accessdata.fda.gov/drugsatfda_docs/nda/2021/213498Orig1s000MedR.pdf.

12. European Medicines Agency. Ponvory: EPAR—Public Assessment Report. 2021. Accessed July 24, 2026. https://www.ema.europa.eu/en/medicines/human/EPAR/ponvory.

13. Hauser SL, Bar-Or A, Cohen JA, et al. Ofatumumab versus teriflunomide in multiple sclerosis. N Engl J Med. 2020;383:546-557.

14. Canadian Agency for Drugs and Technologies in Health. Clinical review report: ofatumumab (Kesimpta). 2021. Accessed July 24, 2026. https://www.cda-amc.ca/ofatumumab.

15. Steinman L, Fox E, Hartung HP, et al. Ublituximab versus teriflunomide in relapsing multiple sclerosis. N Engl J Med. 2022;387:704-714.

16. US Food and Drug Administration. Clinical review: Briumvi (ublituximab-xiiy), BLA 761238. 2022. Accessed July 24, 2026. https://www.accessdata.fda.gov/drugsatfda_docs/nda/2022/761238Orig1s000MedR.pdf.

17. Montalban X, Vermersch P, Arnold DL, et al. Safety and efficacy of evobrutinib in relapsing multiple sclerosis (evolutionRMS1 and evolutionRMS2): two multicentre, randomised, double-blind, active-controlled, phase 3 trials. Lancet Neurol. 2024;23:1119-1132.

18. Oh J, Bar-Or A, Giovannoni G, et al. Efficacy and safety of fenebrutinib vs teriflunomide in relapsing multiple sclerosis: results of the FENhance 1 and 2 studies. Presented at: American Academy of Neurology Annual Meeting; April 18-22, 2026; Chicago, IL, USA, and virtual.

19. Miller AE, O'Connor P, Wolinsky JS, et al. Pre-specified subgroup analyses of a placebo-controlled phase III trial (TEMSO) of oral teriflunomide in relapsing multiple sclerosis. Mult Scler. 2012;18:1625-1632.

20. Turner B, Cree BAC, Kappos L, et al. Ocrelizumab efficacy in subgroups of patients with relapsing multiple sclerosis. J Neurol. 2019;266:1182-1193.

21. Hartung HP, Robertson D, Steinman L, et al. Efficacy of ublituximab in people with highly active relapsing multiple sclerosis. Neurol Ther. 2026;15:1081-1097.

22. Alvarez E, et al. Ublituximab efficacy in highly active relapsing multiple sclerosis: ULTIMATE I and II subgroup analysis. Presented at: American Academy of Neurology Annual Meeting; 2023. Public conference poster.

23. Kappos L, et al. Early treatment with ponesimod in relapsing multiple sclerosis: subgroup analyses from OPTIMUM. Presented at: Consortium of Multiple Sclerosis Centers Annual Meeting; 2022. Public conference poster.

24. F Hoffmann-La Roche Ltd. Ocrelizumab for the treatment of multiple sclerosis: application for inclusion in the WHO Model List of Essential Medicines. 2023. Accessed July 24, 2026. https://cdn.who.int/media/docs/default-source/essential-medicines/2023-eml-expert-committee/applications-for-addition-of-new-medicines/a28_ocrelizumab.pdf?sfvrsn=44e19aef_2.

25. European Medicines Agency. Ocrevus: European Public Assessment Report. Marketing authorisation issued Jan 8, 2018. Accessed July 24, 2026. https://www.ema.europa.eu/en/medicines/human/EPAR/ocrevus.

26. Genentech. FDA approves Genentech's Ocrevus (ocrelizumab) for relapsing and primary progressive forms of multiple sclerosis. March 28, 2017. Accessed July 24, 2026. https://www.gene.com/media/press-releases/14657/2017-03-28/fda-approves-genentechs-ocrevus-ocrelizu.

27. F Hoffmann-La Roche Ltd. Roche reports a strong start in 2018. April 26, 2018. Accessed July 24, 2026. https://assets.cwp.roche.com/imported/en/20180426_Q1_sales_2018_en.pdf.

28. Henderson M, Horton DB, Bhise V, Pal G, Bushnell G, Dave CV. Initiation patterns of disease-modifying therapies for multiple sclerosis among US adults and children, 2001 through 2020. JAMA Neurol. 2023;80:860-867.

29. Murad MH, Wang Z. Guidelines for reporting meta-epidemiological methodology research. Evid Based Med. 2017;22:139-142.

30. US Food and Drug Administration. Medical review: Aubagio (teriflunomide), NDA 202992. 2012. Accessed September 23, 2026. https://www.accessdata.fda.gov/drugsatfda_docs/nda/2012/202992Orig1s000MedR.pdf.

31. ClinicalTrials.gov. ULTIMATE II protocol, NCT03277248. Accessed September 23, 2026. https://cdn.clinicaltrials.gov/large-docs/48/NCT03277248/Prot_000.pdf.

32. ClinicalTrials.gov. evolutionRMS 1 protocol, NCT04338022. Accessed September 23, 2026. https://cdn.clinicaltrials.gov/large-docs/22/NCT04338022/Prot_000.pdf.

33. ClinicalTrials.gov. GEMINI II protocol, NCT04410991. Accessed September 23, 2026. https://cdn.clinicaltrials.gov/large-docs/91/NCT04410991/Prot_000.pdf.

34. Sanofi. Tolebrutinib clinical trial program update. August 8, 2022. Accessed September 23, 2026. https://www.sanofi.com/en/media-room/press-releases/2022/2022-08-08-17-08-40-2494173.

35. Canadian Agency for Drugs and Technologies in Health. Detailed outcome data: teriflunomide (Aubagio). Accessed September 23, 2026. https://www.ncbi.nlm.nih.gov/books/NBK253627/.

36. European Medicines Agency. Aubagio: product information. Accessed September 23, 2026. https://www.ema.europa.eu/en/documents/product-information/aubagio-epar-product-information_en.pdf.
